# Cross-Species Genome Wide Expression Analysis during Pluripotent Cell Determination in Mouse and Rat Preimplantation Embryos

**DOI:** 10.1371/journal.pone.0047107

**Published:** 2012-10-15

**Authors:** Elisa A. Casanova, Michal J. Okoniewski, Paolo Cinelli

**Affiliations:** 1 Institute of Laboratory Animal Science, University of Zurich, Zurich, Switzerland; 2 Functional Genomics Center Zurich, University of Zurich and ETH Zurich, Zurich, Switzerland; 3 Center for Applied Biotechnology and Molecular Medicine, University of Zurich, Zurich, Switzerland; 4 Division of Trauma Surgery, Center for Clinical Research, University Hospital Zurich, Zurich, Switzerland; University of Newcastle upon Tyne, United Kingdom

## Abstract

The transition between morula and blastocyst stage during preimplantation development represents the first differentiation event of embryogenesis. Morula cells undergo the first cellular specialization and produce two well-defined populations of cells, the trophoblast and the inner cell mass (ICM). Embryonic stem cells (ESCs) with unlimited self-renewal capacity are believed to represent the *in vitro* counterpart of the ICM. Both mouse and rat ESCs can be derived from the ICM cells, but their *in vitro* stability differs. In this study we performed a microarray analysis in which we compared the transcriptome of mouse and rat morula, blastocyst, and ICM. This cross-species comparison represents a good model for understanding the differences in derivation and cultivation of ESCs observed in the two species. In order to identify alternative regulation of important molecular mechanisms the investigation of differential gene expression between the two species was extended at the level of signaling pathways, gene families, and single selected genes of interest. Some of the genes differentially expressed between the two species are already known to be important factors in the maintenance of pluripotency in ESCs, like for example *Sox2* or *Stat3*, or play a role in reprogramming somatic cells to pluripotency like *c-Myc*, *Klf4* and *p53* and therefore represent interesting candidates to further analyze *in vitro* in the rat ESCs. This is the first study investigating the gene expression changes during the transition from morula to blastocyst in the rat preimplantation development. Our data show that in the pluripotent pool of cells of the rat and mouse preimplantation embryo substantial differential regulation of genes is present, which might explain the difficulties observed for the derivation and culture of rat ESCs using mouse conditions.

## Introduction

**Figure 1:**
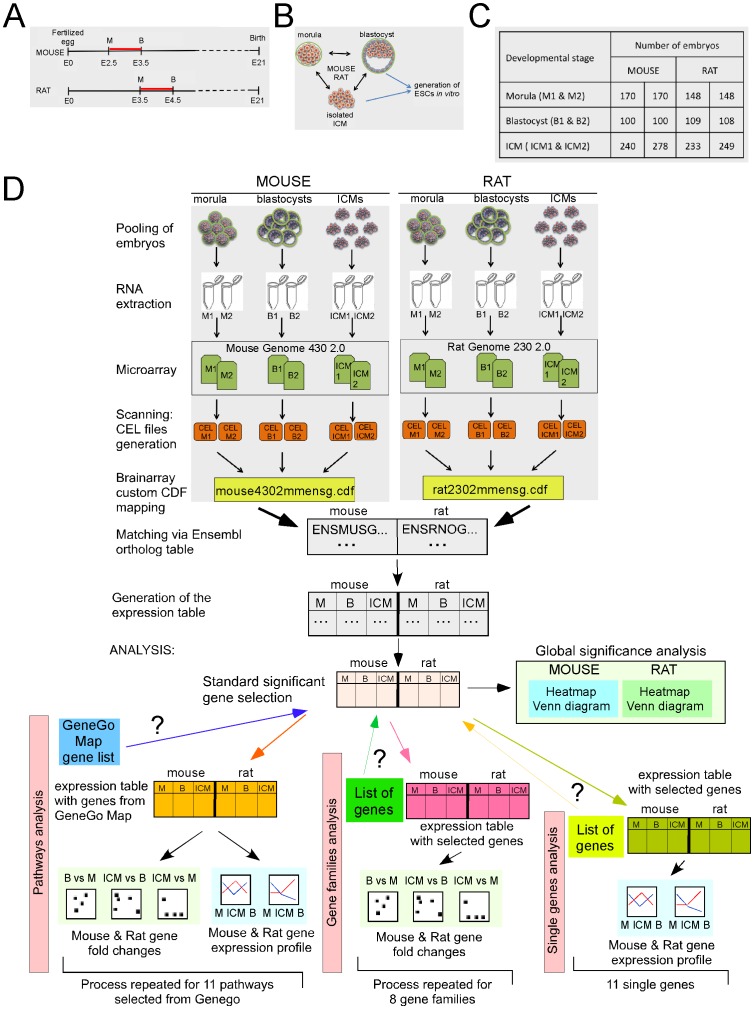
Mouse and rat late preimplantation embryo development: a cross species analysis. **A.** Schema of mouse and rat embryo development length. M: morula stage embryo; B: blastocyst stage embryo. E: embryonic day. **B.** Schema of the three cell populations used for the cross species analysis of gene expression. The blastocyst embryo consists in the inner cell mass (ICM) and in the trophoblast cells. Embryonic stem cells (ESCs) are derived *in vitro* from the ICM cells. **C.** Numbers of embryos collected for the whole genome expression analysis. **D.** Schematic overviews of the screening strategy used in this study. M1 and M2: pool 1 and pool 2 of morula stage embryos; B1 and B2: the two pools of blastocysts; ICM 1 and ICM2: the two pools of isolated ICMs. B vs M: blastocyst versus morula; ICM vs B: ICM versus blastocyst; ICM vs M: ICM versus morula.

The period of time that lasts from the fertilization of the egg to the implantation of the blastocyst represents an attractive model for studying regulatory networks that determine cell fate decisions. Of particular interest is the transition between morula and blastocyst stages, which is the period where pluripotent cells are formed. Morula cells undergo the first cellular specialization and produce an outer rim of cells, the so-called trophoblast that surrounds an inner core of cells the inner cell mass (ICM). The signals that regulate differentiation of the trophectoderm are largely unknown. One of the key discoveries of the last century was the observation that after transferring blastocyst stage embryos in an artificial context it is possible to establish cells, which retain the pluripotent state. These cells, also known as embryonic stem cells (ESCs) are derived from the ICM of the blastocysts [1], [2] and exhibit unique characteristics: They unlimitedly self-renew *in vitro* and are able to contribute to the formation of all cells of an adult organism. Understanding how this population of cells is formed and maintained is of fundamental importance not only for developmental biology but also for regenerative medicine and cancer biology. Nowadays, ESCs are routinely derived from mouse blastocyst embryos, even though not with any difficulties. The mouse has represented for many years the sole organism where pluripotent and germline competent ESCs could be derived. Only recently, almost 30 years after the establishment of the first mouse ESC line, genuine rat ESCs have been generated [3], [4]. The real identity and stability of these cells is not yet completely understood, especially because mouse ESCs and rat ESCs are derived and cultivated under different conditions. Mouse ESCs can be maintained in medium containing inhibitors of the fibroblast growth factor (FGF)/mitogen-activated protein kinase (MEK)/extracellular signal-related kinase (ERK1/2) and of the glycogen synthase kinase 3 (GSK3). These culture conditions are known as the 3i or 2i culture conditions [5] and have been also used for the successful establishment of mouse ESCs from non-permissive mouse strains such as the non-obese diabetic (NOD) mice [6]. Molecularly, rat ESCs express the same pluripotency markers like mouse ESCs [3], [4] but can be established and maintained *in vitro* only under defined culture conditions and additionally in the presence of LIF and feeders. The difference between the two species is also mirrored at the preimplantation development level. Mouse embryos reach the blastocyst stage at day E3.5 whereas the rat at day E4.5 (Figure 1A), nevertheless both species give birth at day E21. These differences highlight the complexity of the mechanisms that define the pluripotent state of a cell and let to assume that in the rat other molecular mechanisms might be involved in the maintenance of the pluripotent state *in vitro* compared to the mouse.

Extending the knowledge of the molecular processes driving the establishment of pluripotency *in vivo* is decisive for understanding the identity and properties of ESCs *in vitro*. We therefore reasoned that a comparison of the gene expression profiles in preimplantation embryos in the mouse and in the rat would be of advantage for improving the comprehension of the pluripotent state and eventually for optimizing derivation and cultivation of rat ESCs. With this purpose we examined and compared with a molecular genetic approach the global gene expression in morula, blastocyst, and in isolated ICM of mouse and rat. With this cross species gene expression comparison we were able to highlight different regulation not only of important developmental pathways like Wnt and Notch, but also of genes known to play important roles in the maintenance of pluripotency in ESCs and in reprogramming processes like for example *Sox2*, *Klf4*, *c-Myc* and *p53*.

## Results and Discussion

### Statement of Grounds and Experimental Design

**Figure 2:**
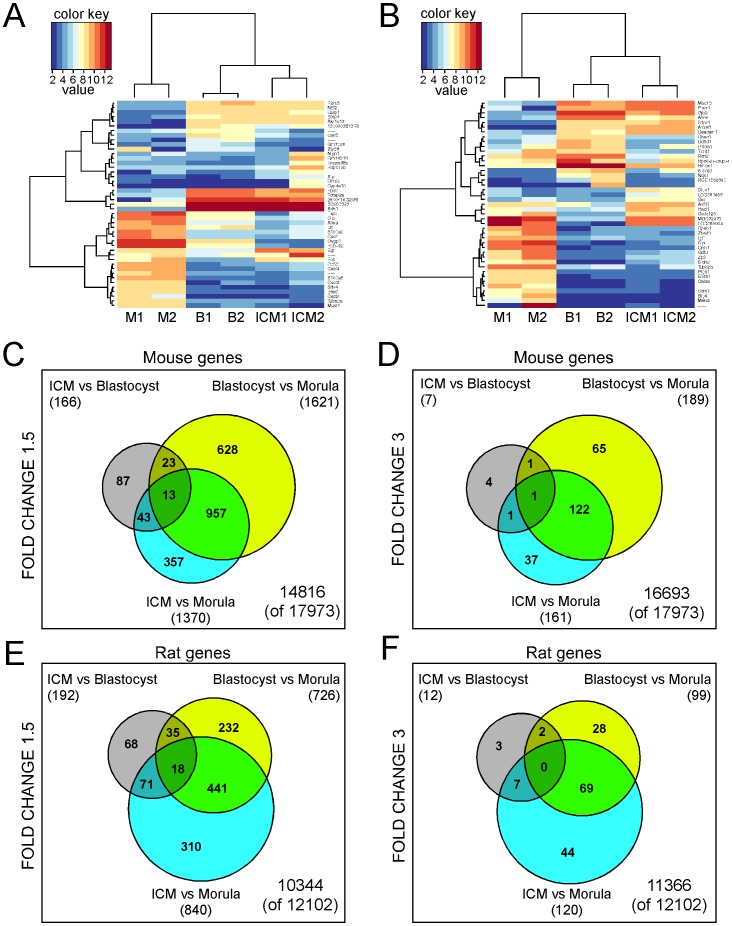
Global significant analysis. **A.** Mouse **B.** Rat heatmaps. The genes are selected by the highest fold change –20 genes in each pair wise comparison of sample means. M1 and M2: morula samples 1 and 2; B1 and B2: blastocyst samples 1 and 2; ICM1 and ICM2: isolated inner cell mass (ICM) cells samples 1 and 2. **C–F.** Venn diagrams of the overlap of mouse and rat genes with significant differential expression (fold change higher than 1.5 and higher than 3) in the three comparisons: ICM versus (vs) Blastocyst, Blastocyst versus Morula, and ICM versus Morula.

During early embryogenesis, pluripotency is a characteristic property of a distinct number of cells of the morula and the ICM of the blastocyst, from where pluripotent ESCs are established (Figure 1B). We collected morula and blastocysts stage embryos from mouse and rat and, by immunosurgery, we isolated the ICM cells from the blastocysts. All the embryos and ICMs were pooled into two groups for every developmental stage (Figure 1C). Pooling of embryos for RNA extraction in this study was chosen mainly because of the low amounts of RNA that can be isolated from preimplantation embryos, and in addition because of the heterogeneity of the cell populations present in the embryos. For the analysis we pooled a large number (n>100) of the independent isolated embryos to achieve a sufficient accuracy of biological pooling (Figure 1C). Due to the difficulties to isolate a larger number of embryos from mice and rats, we performed the microarray study by using two replicate samples per developmental stage (Figure 1D). The global significance analysis of the mouse and rat expression profiles is depicted on the Figure 2A and 2B. Top 20 differentially expressed probe sets for the mouse (Figure 2A) and for the rat (Figure 2B) have been selected in each of the pairs of treatments and then used at the input of hierarchical clustering for the heatmap. The heatmap shows that each pairwise comparison has a group of upregulated and downregulated genes, however on the global level there is hardly any overlap in terms of orthologs (just one gene in common in the heatmap built with over 50 genes from the three top 20 lists). This leads to the conclusion that prior biological knowledge should be used for the search of meaningful relationships. We therefore gathered the information present in the GeneGo pathways in order to investigate the similarities and differences locally, within the context of pathways and gene families.

### Identification of Differentially Expressed Genes in the Three Cell Populations

To reveal similarities and differences in the regulatory mechanisms controlling mouse and the rat development of morula and blastocyst, we first analyzed the data of the mouse and the rat microarray study separately. We selected the genes that had a fold change (understood as the difference between log2 signal values) higher than 1.5 in the three comparisons (see Table S1A–C and S2A–C): ICM versus blastocyst (ICM vs B), blastocyst versus morula (B vs M), and ICM versus morula (ICM vs M). For the mouse study we found that out of 17′973 genes 166 were differentially regulated between the ICM and the blastocysts (Figure 2C and Table S1A). A higher number of differentially regulated genes was found in the comparison B vs M, where 1′621 genes had a fold change higher than 1.5 (Figure 2C and Table S1B). In the comparison ICM vs M we found 1′370 genes, 957 of which were also differentially expressed between blastocyst and morula (Figure 2C and Table S1C). Between the 957 genes found in both the comparisons ICM vs M and B vs M a clear upregulation of the transcription factor *Stat3* and the Lif receptor *Lifr* were present. Both genes were upregulated in the blastocyst or the in ICM compared to the morula but were not differentially expressed in the comparison ICM vs B (Table S1B and S1C), confirming their specific expression in the blastocyst. This is interesting because previous data suggested that the LIF/STAT3 pathway is dispensable during the preimplantation embryo development in the absence of diapause [7]. Nevertheless, this pathway plays a fundamental role *in vitro* in the maintenance of pluripotency and derivation of ESCs [8], [9], [10]. Our data highlight the possibility that the characteristic expression of these genes at the blastocyst stage might indeed play an important role, and that the previously performed studies with knockout embryos could have been biased by the induction of compensatory mechanisms.

Only 23 genes were differentially expressed in both the comparisons ICM vs B and B vs M (Figure 2C). Between them, the *Celf5* gene, a member of the CELF gene family [11], showed a 3.5 fold upregulation in the comparison B vs M and a −2.5 fold downregulation in ICM vs M, indicating a potential function in the trophoblast cells of the blastocyst.

Aiming at the identification of genes that are very characteristically expressed in the ICM, we have initially used the strict threshold on the fold changes (absolute log2 FC >3, see Figure 2D) The gene *Fos* had a fold change of 3.5 in the comparison ICM vs B and −3.9 in the comparison B vs M (Table S1A and S1B) indicating that *Fos* expression is high in the morula and persists in the ICM cells of the blastocyst. Interestingly, it has been shown that *Fos* is also expressed *in vitro* in undifferentiated ESCs and disappears as soon as the cells undergo differentiation [12]. A second gene with characteristic ICM expression is *Egr1*, which was upregulated 4 times in the comparison ICM vs B and was strongly downregulated in the comparison B vs M (Table S1C) suggesting a specific role in the mouse ICM. For the rat study we identified 192 out of 12′102 genes that had a fold change higher than 1.5 in the comparison ICM vs B (Table S2A), among these 71 were also found differentially regulated in the comparison ICM vs M (Figure 2E) and 7 of them showed a fold change higher than 3 (Figure 2F). The genes *Nqo1*, *Ddhd1*, *Hmox1* and *Chac1* had a positive fold change in both the comparisons ICM vs B and ICM vs M (Table S2C), indicating that they are upregulated exclusively in the ICM cells of the rat blastocyst. None of these 7 genes was found in the mouse study in the comparison ICM vs B and ICM vs M, except for the *Nqo1* (NAD(P)H quinone oxidoreductase) that was upregulated in the ICM compared to the morula, nevertheless with a factor of 1.5. It has been shown that inhibition of NQO1 causes degradation of p53 in various cell types [13] therefore NQO1 supports the accumulation of p53, which leads to the induction of growth arrest [14], [15] and/or apoptosis [16].

In a second step we performed a global analysis of these datasets with the GeneGo software using Metacore annotation database to assign functional biological processes to each individual species dataset. For every comparison we selected the 20 more significant processes present in the 1.5 fold change gene groups (Figure S1). This analysis highlighted that in the three comparisons there are different biological processes taking place in the two species. Seen that the rat preimplantation embryo development is shifted compared to the mouse of about 24 hours, it is reasonable to assume that processes like cell cycle or proliferation differs in the two species at these developmental stages (Figure S1).

In summary, by comparing the gene expression in the morula and blastocyst from the mouse and from the rat, we demonstrated that there are differential regulations of factors between the two species. Further analyses are needed in order to understand if these genes could have a function in the establishment of ESCs. Of special interest are those upregulated in the comparison ICM vs B in the mouse and in the rat, because they might represent new factors involved in the establishment and maintenance of the ICM cells, and therefore they might be as well critical factors in the ESCs.

### Cross Species Analysis of Selected Pathways

The purpose of this study was to identify molecular pathways or genes, which are differentially expressed between the mouse and the rat, in order to gain insight into the molecular processes governing pluripotency in the rat. We analyzed fold changes between mouse and rat in 11 selected pathways from GeneGo (GeneGo Maps). A list of all the genes and the selected pathways as well as the gene fold changes is reported in Table S3. For every comparison (B vs M, ICM vs B, and ICM vs M) we generated a plot comparing the fold change value of the selected genes in the mouse with the ones of the same gene in the rat. Every dot represents a gene. Red dots correspond to genes that have similar fold change values between the two species. Green dots label genes with different fold change values between rat and mouse in the selected comparison. Interesting genes are highlighted with a special label that allows following the expression through all the three comparisons. With this representation is possible to get an overview on genes which show either similar or differential expression trends in the two species. A list of the genes showing the most significant differences is available in Table S5.

#### The Notch pathway

**Figure 3:**
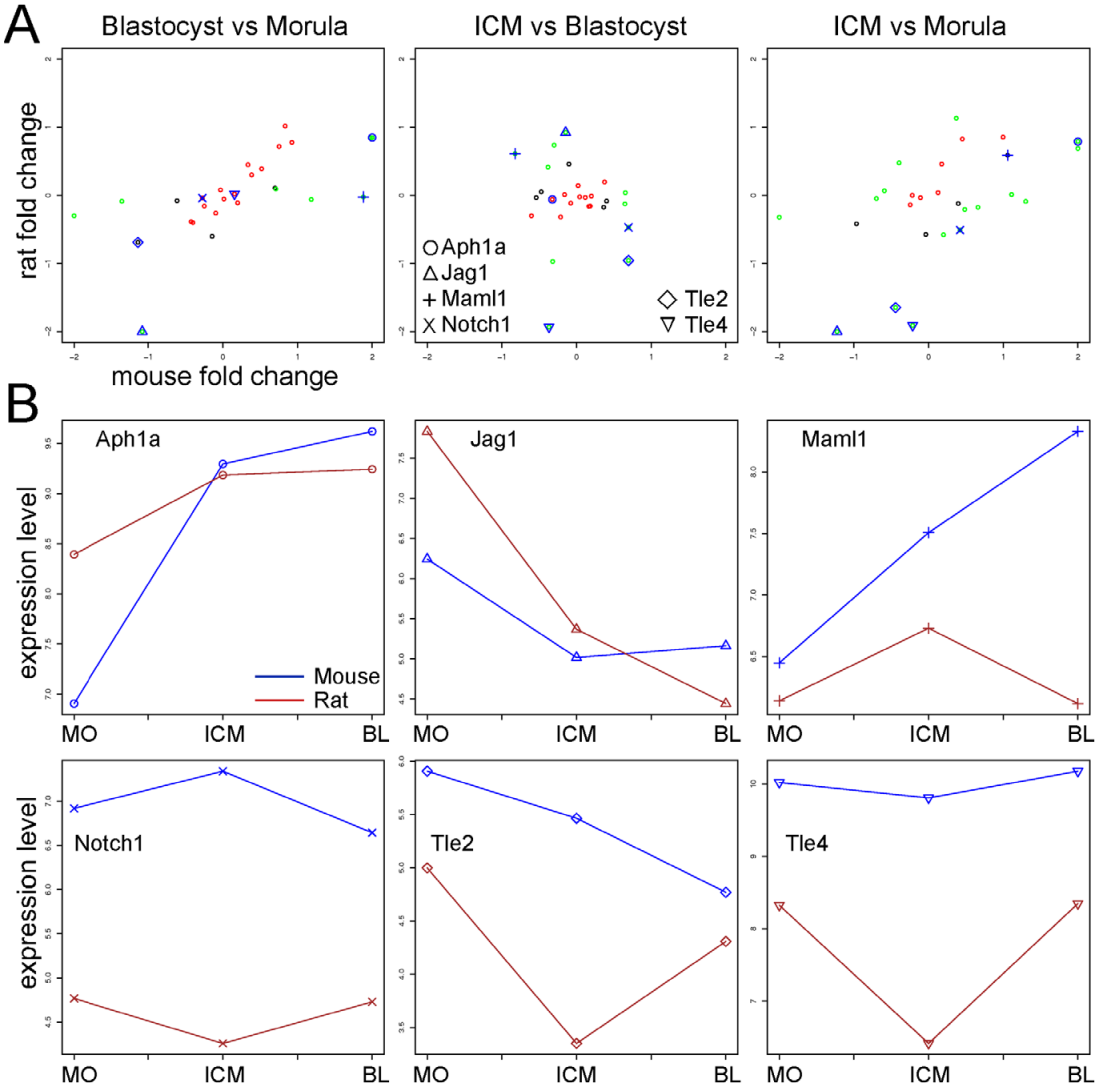
**A.** Cross species analysis of the genes in the Notch pathway. Fold change scatterplots. Cross species comparison of the fold change expression of the genes in the pathway “Development, Notch Signaling Pathway” from GeneGo. In green are marked the genes for which fold change differ in mouse and rat within the three comparisons: Blastocyst versus (vs) Morula, ICM versus Blastocyst, and ICM versus Morula. In red are marked those genes that have a similar fold change pattern in the two species in each comparison. With a special marker there are highlighted 6 selected genes that have differential expression patterns in the two species. The complete list of all the genes analyzed with their fold changes is reported in Table S3. **B.** Expression signal profile plots. Expression level of 6 selected genes from the Notch pathway. In blue are marked the expression levels of the genes in the mouse and in red the one in the rat embryos. MO: Morula, ICM: Inner cell mass, BL: Blastocyst. The unit is log2 of measured expression.

The Notch pathway is a highly conserved cell signaling system present in most multicellular organisms; it influences differentiation, proliferation, apoptotic events at all stages of development, and importantly it has also been implicated in different aspects of stem cell biology [17]. Notch controls the cell fate choices depending on the differential expression of ligands and receptors in opposing cells. We analyzed 27 genes present in the pathway “Development Notch Signaling Pathway” in GeneGo. In the comparison B vs M most of the 27 genes behaved similar in both species (Figure 3A). Our study however identifies *Notch1*, one of the four known Notch receptors, being upregulated in the mouse and downregulated in the rat in the comparison ICM vs B as well as in ICM vs M (Figure 3A). Thus, *Notch1* is upregulated in the mouse ICM but downregulated in the rat ICM (Figure 3B). Activated Notch1 can promote, depending from the context, either differentiation processes or maintenance of stem cell proliferation (reviewed in [17], [18]). Therefore, due to its variety of molecular functions, the finding that *Notch1* is differentially expressed in the mouse and rat preimplantation embryos, especially in the cells of the ICM, suggests a possible different role of this gene in the two species.

We also observed significant changes in the expression levels of other 5 genes: *Aph1a*, *Jag1*, *Maml1*, *Tle2*, and *Tle4* (Figure 3B). The gene *Aph1a* was upregulated in the mouse but only slightly changed in the rat in the comparison B vs M (Figure 3A and Table S3). This gene encodes the membrane protein APH-1 that is an essential member of the γ-secretase, which cleaves single-pass transmembrane proteins at residues within the transmembrane domain. APH-1a is the major mammalian APH-1 isoform required for Notch signaling during embryogenesis [19].

Interestingly, the Notch ligand *Jag1* was downregulated during the transition from morula to blastocyst stage in both species, but this downregulation was stronger in the rat embryos than in the mouse (Figure 3A). The gene *Maml1* is involved in the regulation of the transcriptional activation of Notch target gene expression. *Maml1* was strongly upregulated in the mouse in the comparison B vs M and ICM vs M (Figure 3A) indicating that Maml1 expression increases from the morula to the blastocyst stage. In the rat however, *Maml1* was specifically upregulated in the cells of the ICM (Figure 3B) highlighting once more a potentially different activation of the Notch pathway in the two species.

The most important targets of the Notch1-complex are the HES genes, which are transcriptional repressors that rely on the general corepressor Groucho/Transducin-like enhancer of split (TLE) protein family [20]. Thus, TLE corepressors represent a key effecter of the Notch pathway. We found that in the comparison B versus M, *Tle2* and *Tle4* have a similar expression patterns in the rat and in the mouse (Figure 3A). However, in both the comparisons ICM vs B and ICM vs M *Tle2* and *Tle4* were downregulated in the rat, indicating a specific downregulation in the ICM cells (Figure 3B). These findings are in agreement with the observation that Maml1, the regulator of the transcriptional activation of Notch target gene expression is upregulated in the rat ICM. Of interest is that Notch1 and its ligands, Jagged1, Jagged2, and Delta3, are known to be expressed in mouse ES cells. Overexpression of Notch does not alter the stem cell phenotype in the presence of self-renewal stimuli, but upon their withdrawal, differentiation is directed exclusively towards the neural lineage [21].

These data clearly show that the control of the regulation of the Notch pathway components in mouse and rat occurs at different levels. In the mouse where the expression of Notch1 is relatively high the regulation occurs by activation of inhibitory components like Maml1 and Tle2/4 whereas in the rat the pathway is transcriptionally inactive. It could therefore be important in order to enhance the efficiency of rat ESC derivation to inhibit the Notch pathway activity.

#### Analysis of regulators of the cell cycle

As previously mentioned there are strong differences during the preimplantation development of mouse and rat embryos. Mouse embryos need around three days to reach the blastocyst stage, what leads to a mean cell division time during this period of about 14 h [22]. In reality every cell division cycle during the preimplantation development has different lengths (reviewed by [23]). Of especial importance is the generation at the morula stage of blastomeres, which differ in size and cell division dynamic, and at the blastocyst stage they differentiate into trophoblast and the ICM cells. A typical characteristic of ESCs, isolated from the ICM, is that they exhibit an exceptional cell cycle distribution, where the S phase represents about 75% of the total cell cycle and the G1 phase last for about 1 h [24]. In the rat the formation of the blastocyst is almost 24 h delayed compared to the mouse, the reason why the rat blastomeres are dividing slower than the mouse ones is largely unknown. In order to elucidate the events linked with cell cycle progression in both species we analyzed 11 genes of the GeneGo pathway “Cell cycle Influence of Ras and Rho proteins on G1/S Transition” that clearly showed differential expression in the three cell populations (Table S3).

**Figure 4:**
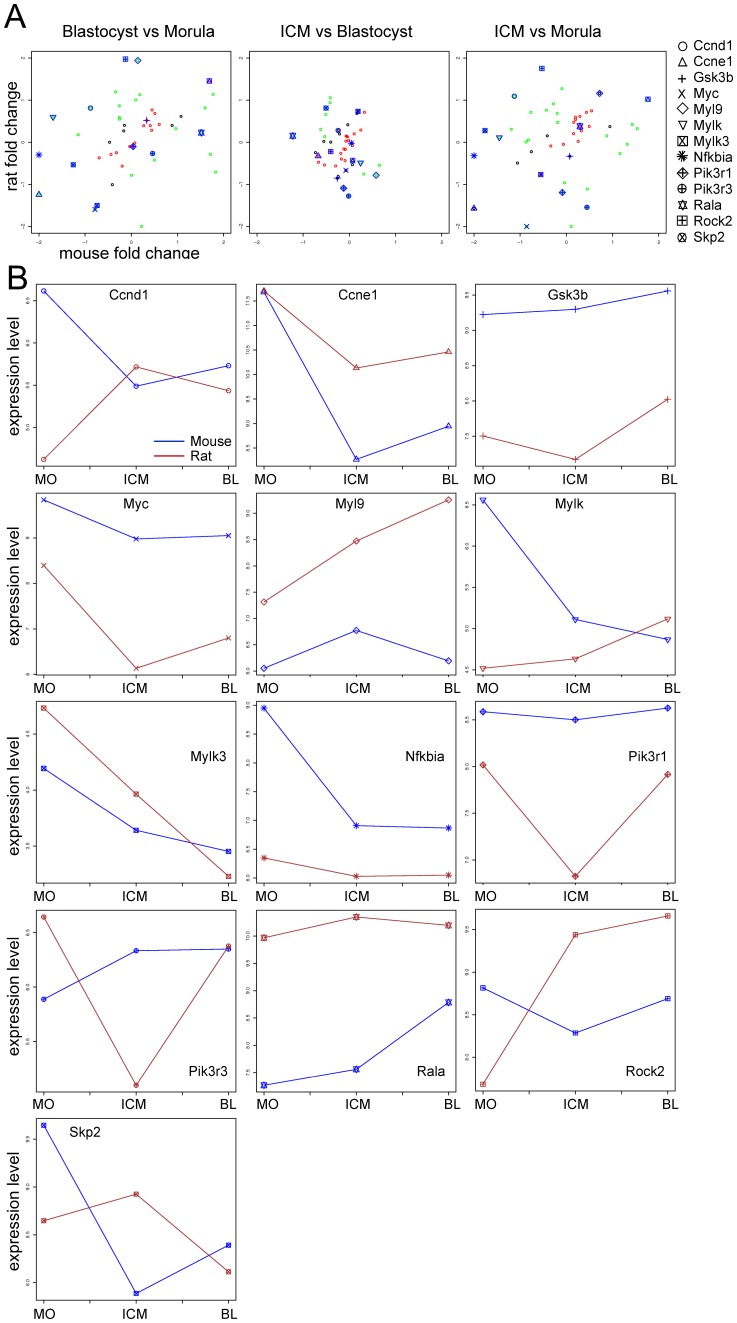
Cross species analysis of cell cycle component. **A.** Fold change scatterplots. Cross species comparison of the fold changes expression of the genes in the pathway “Cell cycle, Influence of Ras and Rho proteins on G1/S transition” from GeneGo (see also Table S3). The data were analyzed as described in Figure 3A. Thirteen genes have been highlighted in order to follow their expression in the three comparisons. **B.** Expression signal profile plots. Expression level of 13 selected genes involved in the regulation of the cell cycle. In blue are marked the expression level of the genes in the mouse and in red the one in the rat embryos. MO: Morula, ICM: Inner cell mass, BL: Blastocyst. The unit is log2 of measured expression.

The gene *cyclin D1* (*Ccnd1*) showed different expression pattern in the mouse and the rat preimplantation embryos. The *Ccnd1* was downregulated for the mouse and upregulated for the rat in both the comparisons B vs M and ICM vs M (Figure 4A). Thus, *Ccnd1* expression in the mouse decreases in the blastocyst and is even stronger reduced in the cells of the ICM compared to the whole blastocyst (Figure 4B). On the contrary *Ccnd1* in the rat embryo is strongly upregulated from the morula to the blastocyst, reaching the highest expression level in the ICM cells (Figure 4B). CCND1, in complex with CDK4/6, phosphorylates during the S phase transition the product of the retinoblastoma (Rb). Rb is involved in the initiation of DNA replication through the activation of E2F, which in turn activates the transcription of cyclin E1 (*Ccne1)* [25]. We observed an upregulation of *Rb* in the mouse for the comparisons B vs M and ICM vs M and a downregulation in the comparison ICM vs B (Figure S2A), indicating an increase in *Rb* expression from the morula stage to the blastocyst stage (Figure S2B). The expression of *Ccne1* in both species showed a similar expression pattern during the development from morula to blastocyst stage embryo (Figure 4B).

Skp2 (S-phase kinase-associated protein 2) is a component of the ubiquitin ligase complex SCF, which is responsible for the ubiquitin-dependent degradation of regulators of the cell cycle. Precisely, Skp2 is involved in the degradation of the Cyclin-dependent kinase (Cdk) inhibitor p27 [26], inducing therefore cell cycle progression. p27 prevents cell cycle progression by inhibiting the Cyclin E-Cdk2 complex formation; in the presence of the Skp2-SCF complex p27 is degraded leading to the activation of the Cyclin E-Cdk2 complex, which causes the entrance into the S phase.

The expression of *Skp2* was for the mouse downregulated in both the comparisons B vs M and ICM vs M (Figure 4A) showing a similar expression trend like *Ccne1*: Downregulation from the morula to the blastocyst stage, with specific low expression level in the ICM cells (Figure 4B). Interestingly, the expression of *Skp2* in the rat was higher in the cells of the ICM (Figure 4B).

During mitosis the cells undergo profound changes in the microfilament structure. The myosin regulatory light chains (Myls) control these morphological changes through their phosphorylation [27], [28]. The phosphorylation of Myls is controlled by the myosin light chain kinases (Mylks). It has been shown that the Rho kinases (ROCK) are also involved in the phosphorylation of Myls [29], [30]. The phosphorylation sites on the Myls vary during the cell cycle progression, inducing their activation or inhibition [31], [32]. Interestingly, the expression of *Myl9, Mylk, Mylk3,* and *Rock2* was differentially regulated within the three comparisons in both species (Figure 4A and 4B), demonstrating once more essential differences between mouse and rat preimplantation development.

c-MYC plays important roles in various physiological processes like cell growth, proliferation, apoptosis, and loss of differentiation [33]. In the comparisons B vs M and ICM vs M *c-Myc* was downregulated in both species, however in a more remarkable manner in the rat (Figure 4A). Interestingly, the expression of *c-Myc* was specifically downregulated in the rat ICM, while it was not changed in the two compartments of the mouse blastocyst (Figure 4B). This is interesting, since *c-Myc* represents an important factor in stem cell biology; moreover it is able *in vitro* in combination with three other transcription factors (*Oct3/4*, *Sox2*, and *Klf4*) to reprogram differentiated cells into pluripotent cells [34]. This expression difference might indicate that eventually an increase of the expression of c-Myc might be necessary for enhancing the establishment of rat ESCs.

Furthermore, in the analysis of the pathway “Cell cycle Influence of Ras and Rho proteins on G1/S Transition” we identified two members of the phosphoinositide-3-kinase pathway (PI3K-AKT): The regulatory subunit 1 (*Pik3r1*) and 3 (*Pik3r3*). Interestingly, in the rat both genes were specifically downregulated in the cells of the ICM (Figure 4A and 4B). The PI3K-AKT pathway has been implicated in many cellular processes like regulation of cell cycle progression, apoptosis, migration, and cell adhesion. We performed the cross species analysis on the pathway “Development Growth hormone signaling via PI3K/AKT and MAPK cascades” from GeneGo (Figure S3A), where we analyzed the expression of *Pik3r1* and *Pik3r3* together with other members of the PI3K-AKT pathway (Figure S3B). The expression of *Gsk3β* was found similarly regulated in both species (Figure 4A). Nevertheless, in the rat *Gsk3β* was specifically downregulated in the cells of the ICM (Figure 4B). It has been shown that authentic rat ESC can be derived and maintained in culture only in the presence of a GSK3β inhibitor [3]. On the contrary, pluripotent mouse ESCs can be established and maintained also under other culture conditions [5], [35], [36]. Our data report a downregulation of *Gsk3β* in the cells of the ICM in the rat but not in the mouse, letting assume that a low level of *Gsk3β* is fundamental in the rat for maintaining the pluripotent state *in vivo* as well as *in vitro*. This could indicate why the use of *Gsk3β* inhibitors is essential for the establishment and cultivation of rat ESCs [3] and rat induced pluripotent stem cells [37], [38]. Optimizing the concentration of GSK3 inhibitors could therefore positively influence the efficiency of generation of pluripotent stem cells in the rat.

Another important signaling that influences the cell cycle is the p53 pathway (Figure S2A and S2B). Interestingly, the gene *p53* (known in the mouse as *Trp53* and in the rat as *Tp53*) was upregulated in the rat in both the comparisons ICM vs M and B vs M (Figure S7A), whereas in the mouse the expression was constant in all the three cell populations (Figure S7B). This could explain why in the rat the gene *Nqo1* (responsible for the degradation of p53) was strongly upregulated in the ICM (Figure 2F and Table S2A). Other genes involved in the regulation of cell proliferation are reported in the Figure S4, where we performed the cross species analysis on the pathway “Development SSTR2 in regulation of cell proliferation” from GeneGo.

During embryo development, the proliferation kinetics of the cells affects their fate determination, so that different cell lineages show faster or longer cell cycle progression [23]. Also in the ESCs *in vitro* a rigorous regulation of the cell cycle is fundamental for the maintenance of pluripotency. This study makes apparent that critical factors involved in the cell cycle and proliferation are differentially expressed in the morula and the blastocyst of mouse and rat. The optimal control of the expression/activity of these genes seems therefore to be essential for the establishment and maintenance of pluripotent ESCs from both rat and mouse. Mouse ESCs cultivated under 2i conditions are composed of a homogenous population of cells expressing the classical pluripotency markers. Interestingly, it was recently shown that when rat ESCs are cultivated under the same conditions in presence of LIF a heterogenous population of cells is present and these cells exhibit differences in the expression of genes that are implicated in cell cycle regulation and in the p53 pathway [39]. This leads to the conclusion that a tight control of the cell cycle is mandatory for obtaining a homogenous population of pluripotent cells in the rat.

#### The TGF and the Wnt signaling

The pathways transforming growth factor β (TGF-β) and Wingless (Wnt) are evolutionary conserved. The transforming growth factor–β (TGF-β) superfamily comprises nearly 30 growth and differentiation factors that include TGF-βs, activins, inhibins, and bone morphogenetic proteins (BMPs). Members of the Nodal/Activin and BMP subfamilies are key players in the generation of axes and in the subsequent patterning of tissues across these axes during embryogenesis (for a review see: [40], [41]). Similarly important are the members of the Wnt pathway, which are active during most developmental stages (for a review see: Kemp et al 2007). Although, their role is not yet clear during the preimplantation development they have been shown to be essential in maintenance of pluripotency in mouse ESCs [42], [43], [44]. Therefore we included this pathway in our cross-species analysis.

**Figure 5:**
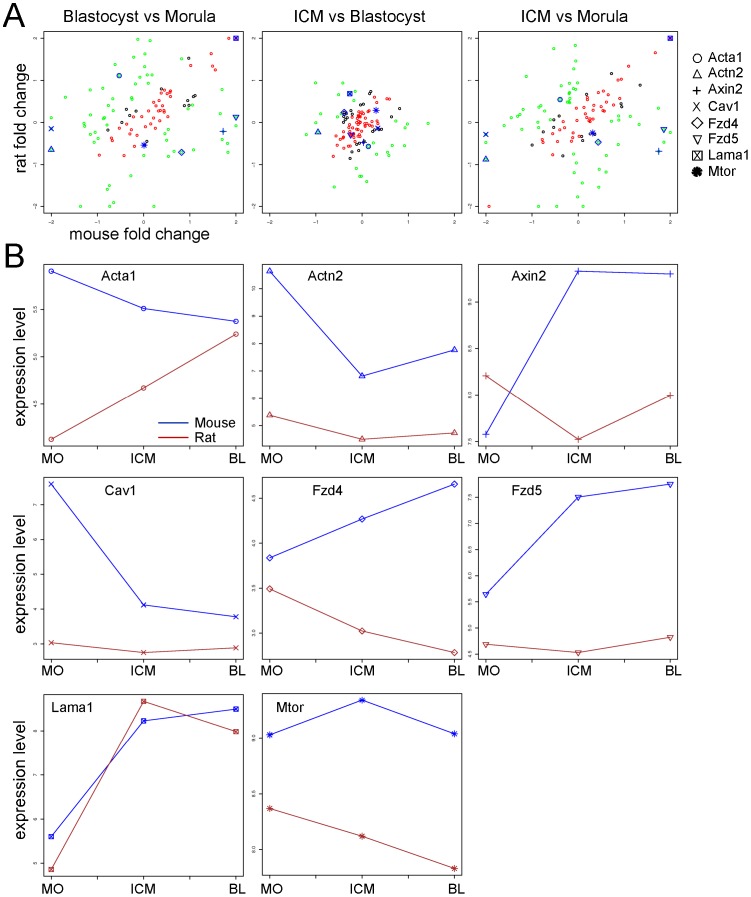
Cross species analysis of the genes in the Wnt and TGF pathways. **A.** Fold change scatterplots. Cross species comparison of the fold changes expression of the genes in the pathway “Cytoskeleton remodeling, TGF, WNT and cytoskeletal remodeling” from GeneGo (see also Table S3). The data were analyzed as described in Figure 3A. Eight genes have been marked with a special label. **B.** Expression signal profile plots. Expression level of the 8 selected genes in the morula, the ICM, and the blastocyst in the mouse (blue) and in the rat (red). MO: Morula, ICM: Inner cell mass, BL: Blastocyst. The unit is log2 of measured expression.

We analyzed 112 genes present in the pathway “Cytoskeleton remodeling TGF, WNT and cytoskeletal remodeling” from GeneGo. We highlighted 8 genes, which had a clear differential expression changes between the three comparisons and between the two species (Figure 5A and 5B).

Wnt signals are transduced depending on their functions through different receptors and members: The canonical Wnt pathway is known to be involved in transmitting signals for cell fate determination, whereas the non-canonical Wnt pathway is involved in controlling cell movements and tissue polarity. The gene *caveolin 1* (*Cav1*) was downregulated in the blastocyst and ICM cells of the mouse (Figure 5A), whereas it was almost not expressed in the rat cells (Figure 5B). Cav1 is an essential component of the caveolae, where it acts as a regulator of caveolae-dependent lipid trafficking and endocytosis [45], [46]. Cav1 can act as a positive as well as a negative regulator of important signaling pathways (reviewed in [47]), for example it negatively regulates the Wnt pathway by recruiting β-catenin and therefore blocking the transcription of the β-catenin target genes [48]. The two membrane receptors *frizzled homolog 4* (*Fzd4*) and *frizzled homolog 5* (*Fzd5*) were upregulated in our analysis in the mouse ICM and blastocyst compared to the morula (Figure 5A). However, in the rat we detected a very low expression of both receptors in all the three cell populations (Figure 5B). This indicates that the Wnt pathway is differentially active in the two species (see also Figure S5). The gene *Axin2* is a downstream target of the Wnt pathway that acts as a negative regulator by directing β-catenin for proteasomal degradation [49]. It has been shown that stable β-catenin and elevated *Axin2* transcription indicates the activation of the Wnt pathway [50]. In our cross species analysis *Axin2* was upregulated in the mouse in both the comparisons B vs M and ICM vs M (Figure 5A), indicating a higher expression in the cells of the blastocyst and of the ICM (Figure 5B). Interestingly, in the rat the expression of *Axin2* decreased specifically in the cells of the ICM (Figure 5B). The three regulators of the Wnt pathway, namely *β-catenin* (Figure S2B), *Axin2* (Figure 5B), and the *Gsk3β* (Figure 4B) had a similar expression pattern in the rat embryos: A reduced expression in the ICM cells compared to the morula and whole blastocyst cells. In the mouse embryos the expression of these three factors was almost constant except for *Axin2* that was upregulated in the ICM and blastocyst compared to the morula. This might indicate that in the rat the Wnt signaling pathway, and especially *β-catenin* could not play a major role in the maintenance of pluripotency in rat ESCs, which is indeed the case for mouse ESCs [42], [43], [44].

Interestingly, these differences are also present in other Wnt- and TGF-pathway genes involved in the apoptotic and survival processes. We analyzed 13 genes from the pathway “Apoptosis and survival NGF signaling pathway” (Figure S6A and S6B) and 20 genes from the pathway “Apoptosis and survival Apoptotic Activin A signaling” (Figure S7A and S7B) from GeneGo. For example the apoptosis related gene *Caspase3* (*Casp3*) was upregulated in the rat in all the three comparisons (Figure S6A) indicating a higher expression in the cells of the blastocyst (Figure S6B). On the contrary in the mouse, *Casp3* was upregulated in the cells of the morula and then the expression decreased in the blastocyst (Figure S6B). Combined with the observation that mouse ESCs lacking the *Casp3* gene show impaired differentiation capacity [51], our data suggest that employing Caspase inhibitors during derivation and cultivation of rat ESCs might be helpful.

### Cross Species Analysis of the Expression Patterns of Selected Gene Families

Based on the genes present on GeneChip® Mouse Genome 430 2.0 arrays and, for the rat on the GeneChip® Rat Genome 230 2.0 arrays, we selected the families of genes. With the same approach used for the analysis of the pathways in the morula and in the blastocyst stage, we further characterized the expression pattern of the genes in the three cell populations for the mouse and for the rat. The complete list of the selected families of genes as well as the fold changes in the three comparisons are listed in Table S4.

#### The BMP-ligands and -receptors family with the intracellular SMADs-family

The bone morphogenetic proteins (BMPs) are members of the transforming growth factor (TGF) super-family and are involved in a variety of processes during embryo development like in the generation and maintenance of organs, in which stem cells play important roles. The signaling pathway starts when the secreted BMP proteins bind to the type I and type II BMP receptors, inducing the activation of the intracellular substrates, the SMAD proteins.

**Figure 6:**
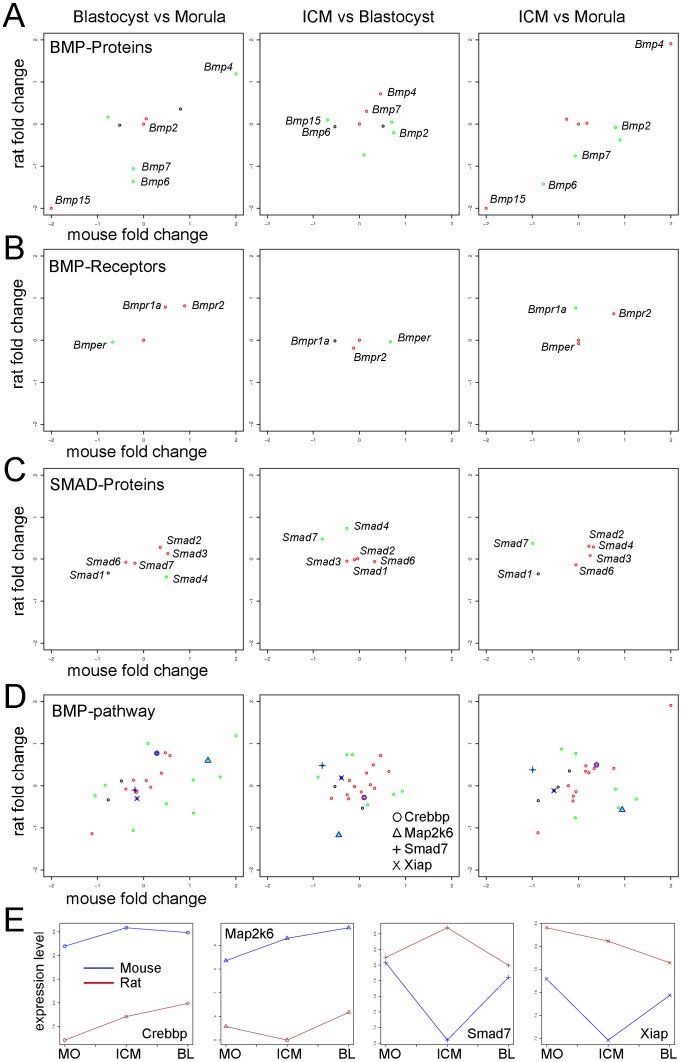
Cross species analysis of regulators of the BMP pathway. **A.** The BMP protein family. Scatterplots of the fold changes measured in the three comparisons for 9 members of the BMP protein family in the mouse and in the rat. The complete list of all the genes analyzed as well as their fold changes are reported in Table S4. **B–C.** Same analysis like for the BMP proteins was performed for 4 members of the BMP receptor family (**B.**) and for 6 members of the SMAD protein family (**C.**). **D.** Fold change scatterplots. Cross species comparison of the fold changes expression of the genes in the pathway “Development, BMP signaling” from GeneGo (see also Table S3). The data were analyzed as described in Figure 3A. **E.** Expression signal profile plots. Expression level analysis of 4 selected genes from the BMP pathway. Mouse: blue; Rat: red; MO: Morula; ICM: Inner cell mass; BL: Blastocyst. The unit is log2 of measured expression.

Here we analyzed the expression of 10 BMP proteins, 4 BMP receptors, and 6 SMAD proteins in the morula, the blastocyst and the isolated ICM, from the mouse and from the rat (Table S4). The genes *Bmp15* and *Bmp4* showed in both species the same expression pattern: Being the former downregulated and the latter upregulated in both the comparisons B and ICM vs M (Figure 6A). This indicates that *Bmp15* is prevalently expressed in the cells of the morula whereas *Bmp4* is upregulated in the cells of the blastocyst and ICM. It is interesting to note that *in vivo* the pluripotent cell population (ICM) of the rat and the mouse has a similar expression of *Bmp4*. It has been shown that *in vitro*, mouse ESCs can be maintained in serum-free culture in the presence of BMP4 or BMP2 in combination with LIF [36]. Nevertheless, withdrawal of LIF and retention of BMP4/2 causes differentiation into epithelial-like cells, leading to the conclusion that the self-renewal response to BMP is dependent on continuous LIF signaling and that the BMP main function is to antagonize the neural differentiation induced by LIF in the absence of serum [36]. All the attempts to derive rat ESCs in serum-containing medium failed in the last years so that nowadays it is possible to establish rat ESCs only under defined, serum-free conditions [3]. Therefore, seen that the expression of *Bmp4* in the ICM of the mouse and the rat blastocyst is similar, it would be interesting to carefully examine the role of BMP4 in rat ESC derivation and maintenance. The expression analysis of other *Bmp-*ligands revealed a general upregulation in the mouse and downregulation in the rat (Figure 6A) whereas no major differences in the two species could be observed for the *Bmp* receptors (Figure 6B). In the comparison ICM vs M the sole gene that was differentially expressed between mouse and rat was *Bmpr1a* that was upregulated in the rat but did not have differential expression in the mouse (Figure 6B).

In the BMP signaling pathway the activated receptors recruit the SMAD molecules, which transmit the signal from the cell surface to the nucleus (Table S4). The expression of the receptor-regulated *Smad1, −2, −3* had a similar expression pattern in both the species in all the three comparisons (Figure 6C). The products of these genes are transcription factors that form complexes with SMAD4 and regulate gene transcription. The expression of *Smad4* increased in the mouse in all the compartments of the blastocyst (Figure 6C) whereas in the rat expression of *Smad4* persisted from the morula to the blastocyst but was specifically upregulated in the cells of the ICM (Figure 6C). In the comparisons ICM vs B and ICM vs M the expression of *Smad7* (inhibitory Smad) was in both cases downregulated in the mouse but upregulated in the rat cells (Figure 6C). It is interesting to notice, that in the mouse we observed an upregulation of the transcription factors *Smad3* and *Smad2* (receptor regulated Smads) in the cells of the ICM and the blastocyst, together with an upregulation of the Co-regulator *Smad4*, whereas the expression of *Smad7* was specifically downregulated in the ICM (Figure 6E).

Analysis of the pathway called “Development BMP signaling” from GeneGo revealed that other genes involved in this pathway are differentially regulated in the morula, ICM, and blastocyst of the mouse and the rat (Figure 6D). The BMP pathway plays important roles in the differentiation of ESCs *in vitro*. Rat ESCs seems to be more sensitive to differentiation stimuli than mouse ESCs, therefore the differential regulation observed *in vivo* of the factors involved in this pathway might reflect also a differential expression *in vitro*, in mouse and rat ESCs.

#### The FGF-factors and FGFR-receptors family

The fibroblast growth factor (FGF) ligands and receptors have been implicated in different phases of the early embryogenesis [52]. The FGF signaling controls proliferation and differentiation of the cells, cell survival, cell morphology and migration, through the activation of important cytoplasmic signal transduction pathways like for example the Ras/ERK pathway and the AKT pathway [53], [54].

**Figure 7:**
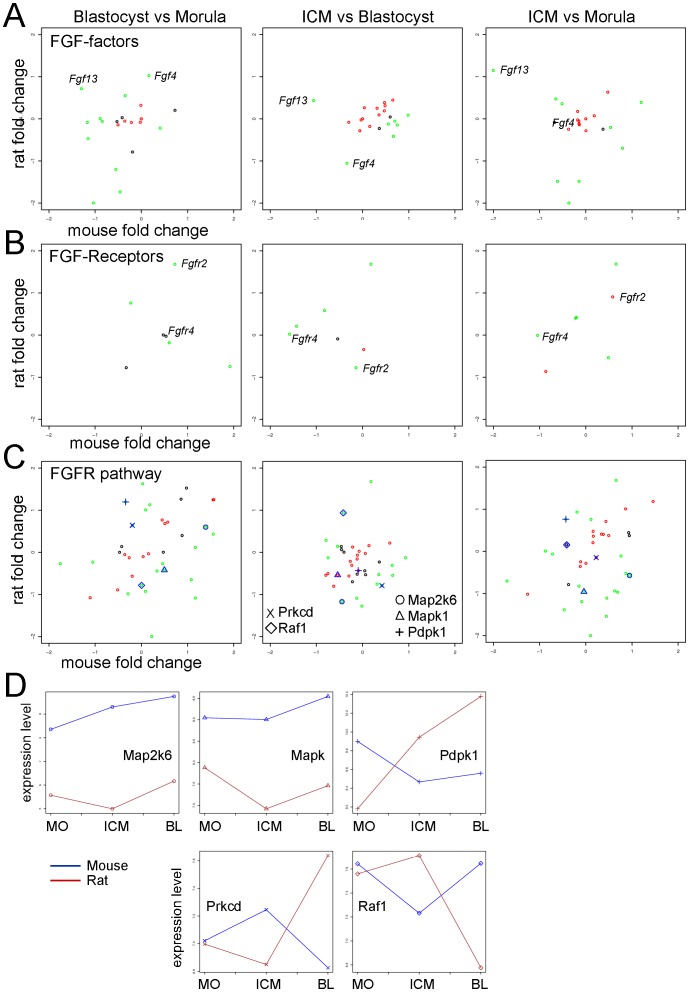
Cross species analysis of regulators of the FGF pathway. **A–B.** Scatterplots of the fold changes measured in the three comparisons for 21 members of the FGF factor family (**A.**) and for 7 FGF receptors (**B.**) in the mouse and in the rat. The complete list of all the genes analyzed as well as their fold changes are reported in Table S4. **C.** Fold change scatterplots. Cross species comparison of the fold changes expression of the genes in the pathway “Development, FGFR signaling pathway” from GeneGo (see also Table S3). The data were analyzed as described in Figure 3A. **D.** Expression signal profile plots. Expression level analysis of 5 selected genes from the FGFR pathway. Mouse: blue; Rat: red; MO: Morula; ICM: Inner cell mass; BL: Blastocyst. The unit is log2 of measured expression.

We analyzed the expression in the three cell populations of 21 FGF factors and 7 cell surface FGF receptors present on the mouse and the rat microarray chip (Table S4). The expression of *Fgf4* was constant in the mouse morula and blastocyst, in the rat embryos however, *Fgf4* expression was upregulated in the comparison B vs M and downregulated in the ICM vs B (Figure 7A). Thus, the expression of *Fgf4* in the rat preimplantation embryo is low in the ICM cells but higher in the trophoblast cells of the blastocyst. This observation is interesting, since rat trophoblast stem (TS) cells are FGF4-dependent [55]. The gene *Fgfr4* was in the mouse downregulated in both the comparisons ICM vs B and ICM vs M, indicating an expression in the morula and trophoblast cells of the blastocyst (Figure 7B), its expression was however not changed in the rat cell populations. The expression of *Fgfr2* increased for both species in the blastocyst, although the upregulation was more predominant in the rat than in the mouse (Figure 7B). The analysis of the pathway “Development FGFR signaling pathway” from GeneGo also highlighted differential expression patterns of genes in the two species (Figure 7C). For example the expression of the gene *Raf1* was similar in the cells of the morula in the mouse and in the rat. However, for the mouse it was downregulated in the ICM cells and upregulated in the whole blastocyst, whereas for the rat it was upregulated in the ICM and downregulated in the whole blastocyst (Figure 7D). Raf1 is a member of the MAPK/ERK pathway (mitogen-activated protein kinase/extracellular receptor kinase), which is stimulated by the FGF factors during embryo development. In a previous study the expression of *Raf1* was detected in both the ICM cells and the trophoblast cells of the mouse blastocyst in a similar amount [56]. We measured however, a downregulation of *Raf1* expression in the ICM cells and an upregulation in the trophoblast cells of the blastocyst. This is in agreement with its involvement in the activation of the FGF signaling that is responsible for the maintenance of the trophoblast cells. Interestingly, the expression of *Raf1* in the rat was downregulated in the trophoblast cells (Figure 7D) and upregulated in the ICM cells, leading to the assumption that this member of the MAPK pathway plays a role in the ICM cells of the rat blastocyst. We further analyzed 13 members of the MAPK family and we found differences in the expression of several genes in the three cell populations of the mouse and the rat (Figure S8A and Table S4). These data suggest that a tight control of the MAPK/ERK pathway members with small chemical compounds might improve the establishment and derivation of pluripotent rat stem cells.

#### The Wnt-ligands family

**Figure 8:**
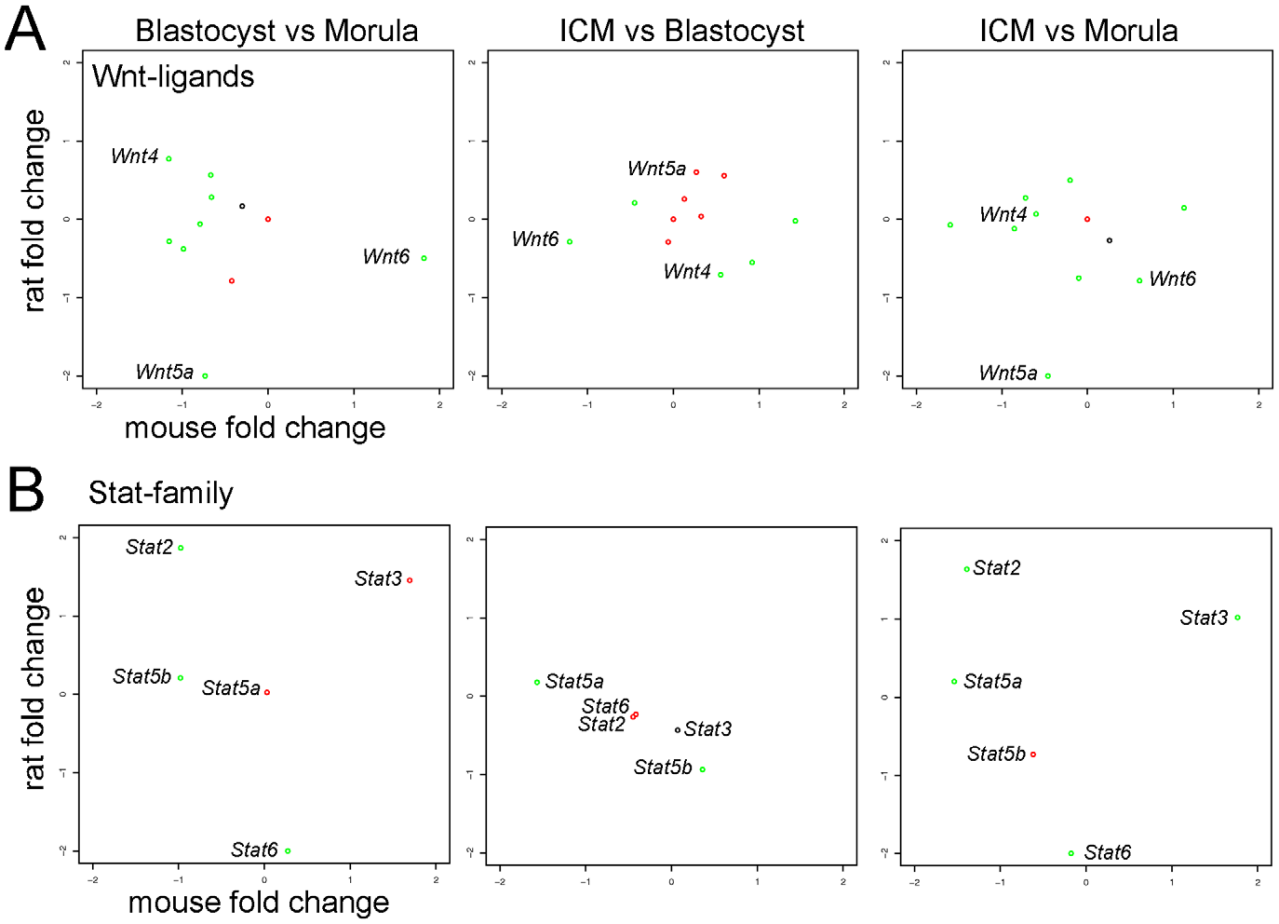
Cross species analysis of the Wnt ligands family and the STAT family. **A–B.** Scatterplots of the fold changes measured in the three comparisons for 11 members of the Wnt family (**A.**) and for 5 STAT family members (**B.**) in the mouse and in the rat. The complete list of all the genes analyzed as well as their fold changes are reported in Table S4.

We have already reported some important changes in the expression of members of the Wnt pathway (Figure 5 and Figure S5). Here we analyzed 17 members of the Wnt-secreted factors and interestingly, we observed that the expression of many Wnt genes is differentially regulated in the mouse and in the rat (Table S4). For example *Wnt6* was upregulated in the trophoblast cells of the mouse blastocyst whereas it was upregulated in the cells of the morula in the rat embryos (Figure 8A). The opposite expression pattern was observed for the gene *Wnt4*, that was upregulated in the mouse in the morula and in the rat in the blastocyst cells. Interestingly, in the rat *Wnt5a* was highly expressed in the cells of the morula and in a lesser extend in the ICM cells (Figure 8A), whereas in the mouse its expression showed only minor differential regulation between the three comparisons (Table S4). The role of the Wnt5a ligand has been extensively studied since it acts through both the canonical and non-canonical Wnt pathway [57]. Importantly, the canonical Wnt pathway has been implicated in the maintenance of pluripotency in mouse ESCs. The WNT5A ligands, together with WNT6, WNT3, and WNT3A were reported to be sufficient for maintaining mouse ESCs in an undifferentiated state in the absence of LIF [58]. Although the precise mode of action of the Wnt pathway in maintaining pluripotency in ESCs needs still to be clarified, it is important to note that factors like *Wnt5a* and *Wnt6* are differentially regulated in the mouse and in the rat in the pluripotent cell compartment of the blastocyst (Figure 8A and Table S4). Further studies will be necessary for clarifying the respective function of these genes in the establishment of the pluripotent cells during preimplantation development.

#### The Stat family

The Signal transducer and activator of transcription (STAT) proteins are cytoplasmic transcription factors that transmit the information received from the transmembrane receptors directly to the nucleus of the cells, where they target the promoter of genes involved in survival, proliferation, and differentiation [59]. Here we analyzed the expression of 5 members of the STAT family (Figure 8B and Table S4). The type I interferons (IFN) are involved in antiproliferative, apoptotic, and antiviral processes, and they are responsible for the activation of STAT1 and STAT2 [60]. In the rat *Stat2* was upregulated in the blastocyst cells, however in the mouse *Stat2* expression decreased from the morula to the blastocyst stage (Figure 8B). The S*tat6* expression was upregulated in the rat in the cells of the morula, whereas it did not show differential expression in the mouse cell populations (Figure 8B).

In the comparison ICM vs B all the *Stats* showed a similar expression in the mouse and in the rat. Only *Stat5a* and *Stat5b* were differentially regulated, being the former higher expressed in the trophoblast cells of the mouse blastocyst whereas the latter was upregulated in the trophoblast cells of the rat blastocyst (Figure 8B). This analysis showed that members of the *Stat* family are differentially regulated in the mouse and rat preimplantation embryos, advising a possible different implication in the development of the morula and blastocyst in the two species. Interestingly in contrast to mouse ESCs, rat ESCs even if derived and cultivated under 2i conditions are LIF dependent. Our data highlights the importance to further analyze the exact role of LIF and other cytokines able to activate STAT-family members during rat development and pluripotent stem cell derivation.

### Expression Pattern Analysis of Genes Related to Pluripotency

The goal of this study was to give a general overview on the regulation of the molecular mechanisms that take place during the development of the mouse and the rat preimplantation embryo form the morula to the blastocyst stage, in order to highlight similarities and differences that could help in the derivation and maintenance of rat ESCs. The LIF/gp130 pathway that leads to the activation of the transcription factor STAT3, plays a fundamental role in the maintenance of pluripotency in mouse ESCs [8], [9], [10], [35], [61] as well as in rat ESCs [3], [4]. Controversially, ESCs show LIF dependence (under certain culture conditions), whereas early epiblast cells do not require LIF stimulation. In fact, *Lif* −/− embryos develop into later stages [7] and embryos carrying mutations on the LIFβR and gp130 receptor develop normally, at least until mid-gestation [62], [63]. Nevertheless, the LIF/STAT3 pathway is indispensable during the preimplantation development, in case of diapause [64]. This observation could explain why embryos do express all the component of this pathway and moreover, why ESCs that are directly derived from the ICM of the blastocyst, are LIF-dependent (reviewed in [65]). Due to the importance of the LIF/gp130-STAT3 pathway in the maintenance of pluripotency in ESCs, we selected 11 genes involved in this pathway and we analyzed their expression in the mouse and rat morula, blastocyst, and ICM.

**Figure 9:**
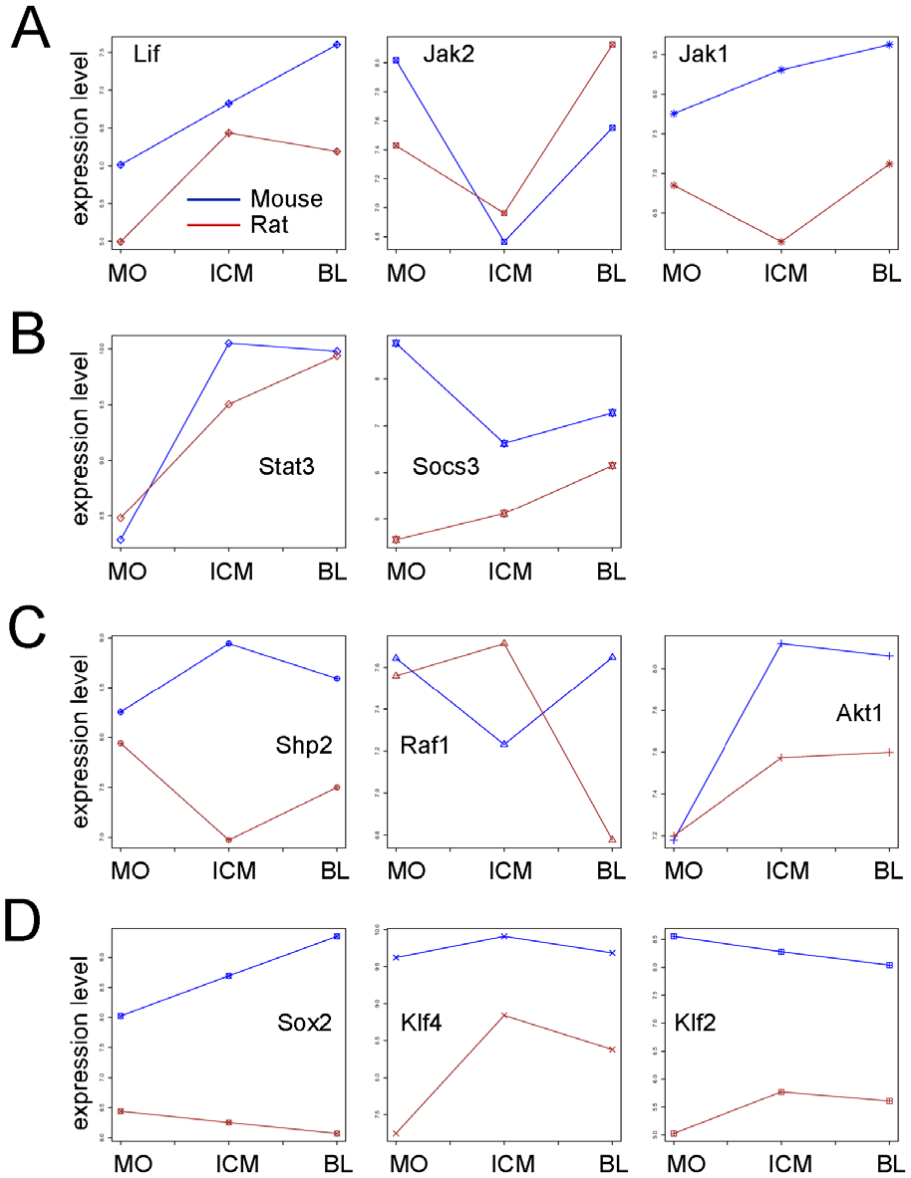
Expression signal profile plots for 11 genes involved in the LIF/gp130 signaling. Expression signal profile plots. **A.** Expression level analysis of *Lif,* which encode the ligand that binds on the LIFβR/gp130 receptor, and *of Jak2* and *Jak1* the receptor-associated Janus Kinases involved in the propagation of the extracellular signaling. **B.** Expression level analysis of *Stat3* and *Socs3*. The transcription factor STAT3 directly controls the transcription of the negative regulator SOCS3. **C.** Expression level analysis of *Shp2*, *Raf1*, and *Akt1.* The products of these three genes lead to the activation of the ERK- and PI3K/AKT-pathways. **D.** Expression level analysis of *Sox2, Klf4,* and *Klf2.* These genes are involved in the maintenance of pluripotency in ESCs. Mouse: blue; Rat: red; MO: Morula; ICM: Inner cell mass; BL: Blastocyst. The unit is log2 of measured expression.

Interestingly, the expression of *Lif* increased in the mouse from the morula to the blastocyst, having a lower expression in the cells of the ICM. On the contrary, in the rat its expression was stable in the ICM cells as well as in the whole blastocyst (Figure 9A). A behavior similar in the two species was observed for *Jak2* that was specifically downregulated in the ICM but upregulated in the blastocyst (Figure 9A). *Jak1* expression indeed, showed in the mouse an analog expression pattern like *Lif*, whereas in the rat it was specifically downregulated in the cells of the ICMs (Figure 9A). The binding of the cytokine LIF to the receptor results in the heterodimerization of the LIFβR and gp130 that causes the activation of receptor-associated JAKs, which are responsible for the phosphorylation and activation of STAT3. JAK1 is necessary for the transmission of the LIF-induced signaling, whereas JAK2 is dispensable. Thus, due to the higher LIF-dependence of rat ESCs in comparison to mouse ESCs, it would be of interest to analyze the expression of *Jak1* in rat ESCs. Interestingly, also the expression of *Stat3* was reduced in the rat ICM cells compared to the whole blastocyst, whereas in the mouse it was constant. Nevertheless, at the morula stage both mouse and rat showed a similar expression level of *Stat3* (Figure 9B). The transcription of the *Socs* genes is directly controlled by STAT3. *Socs3* is responsible for the negative regulation of the LIF/STAT3 signaling [66]. Although we observed a general upregulation in the mouse preimplantation embryo of the components of the LIF pathway, the expression of *Socs3* was downregulated in the ICM and in the whole blastocyst (Figure 9B). Interestingly, in the rat embryos *Socs3* expression increased in a similar manner like *Stat3*, from the morula to the blastocyst stage (Figure 9B) suggesting again that a well-balanced LIF/STAT3 activation is crucial in the rat. This is of importance for the derivation of rat ESCs indicating that despite the need of LIF for their derivation; applying not optimal concentration of this cytokine could reduce the efficiency of establishment. In parallel to the activation of the STAT3 pathway, binding of LIF to the LIFβR/gp130 receptor leads the activation of the mitogen-activated protein kinase (MAPK) and the phosphatidylinositol-3 phosphate kinase (PI3K) pathways. Active gp130 receptor can associate with the protein tyrosine phosphatase SHP-2 [67], which leads to the activation of the kinases RAS/RAF and finally ERK1/2. The expression of *Shp2* was specifically downregulated in the rat ICM cells whereas it was upregulated in the mouse ICM (Figure 9C). However, the expression of *Raf1* had exactly the opposite expression pattern: Downregulated in the mouse ICM cells and upregulated in the rat ICM, indicating a differential expression in both the ICM cells and the trophoblast cells in the two species (Figure 9C). ERK regulates early differentiation processes *in vivo* as well as *in vitro* [6], [68], so that it has been shown that inhibition of this pathway together with the inhibition of GSK3 is sufficient for maintaining pluripotency in ESCs in the absence of LIF [5].

A downstream effector of the PI3K pathway is the serine/threonine protein kinase B (PKB, also known as AKT). AKT has been implicated in many cellular processes like regulation of the cell cycle progression, cell death, adhesion, migration, metabolism and tumorigenesis. In the mouse and in the rat preimplantation embryo we observed a similar expression pattern of *Akt1*, which increased from the morula to the blastocyst stage, although in the mouse the increase was more prominent (Figure 9C).

The genes *Sox2*, *Klf4*, and *Klf2* are involved in ESCs in the maintenance of pluripotency [69], [70], [71]. Moreover, *Sox2* and *Klf4* together with *cMyc* and *Oct3/4* are the four factors used for reprogramming differentiated cells into induced pluripotent stem cells (iPSCs) [34].

SOX2 is a member of the sex-determining region of the Y chromosome-related (SRY-related) high-mobility group (HMG) box (SOX) family of transcription factors. *Sox2* expression is downregulated in cells with restricted developmental potential. We observed an upregulation of *Sox2* expression in the mouse from the morula to the blastocyst stage (Figure 9D). Interestingly, in the rat embryos *Sox2* was expressed at lower levels compared to the mouse; moreover it was slightly downregulated in the blastocyst compared to the morula (Figure 9D).

Some of the *Klf* genes (Krüppel-factors) have been proposed as downstream targets of LIF/STAT3 pathway in ESCs [70]. In our analysis we observed that the expression of *Klf4* increased in the cells of the rat ICM and was downregulated in the whole blastocyst, whereas in the mouse embryos the upregulation of *Klf4* was less strong in the ICM cells (Figure 9D). Also *Klf2* in the rat was upregulated in the ICM and blastocyst but it was downregulated in the mouse blastocyst and ICM cells (Figure 9D). This is interesting since *Klf2* and *Klf4* have been implicated with important pluripotency factors in mouse ESCs [70]. Thus, the fact that they are differentially regulated in the morula and blastocyst from the rat compared to the mouse, could be a contributing factor for the differences observed between mouse and rat ESCs in the derivation efficiency and culture conditions.

The differential expression of these factors can also be of interest for the reprogramming rat somatic cells to pluripotency. Rat iPSCs could be successfully established in 2008 and it could be shown that they can differentiate into all three germ layers in vitro and in vivo [72], [73] and can contribute to generating chimeric rats [37]. This study clearly indicated that rat iPSCs exhibit extensive spontaneous differentiation and only by combining inhibitors of MEK, GSK3β and of the type 1 TGFβ-receptor ALK5 is possible to stabilize the rat iPSCs cultures [37]. The need of the ALK5-inhibitor is interesting because this is in accordance with our observations that bmp4 and smads are differentially regulated between mouse and rat (Figure 6). Of further interest is that these studies were not able to obtain germline competent rat iPSCs. Germline competence could be obtained by combining MEK- and GSK3β-inhibitors with small molecules blocking FGF receptor tyrosine kinases [38]. Also these observations are in accordance with our data showing that the FGF-pathway is differentially regulated in the two species (Figure 7).

### Conclusion

The higher genetic diversity of the rat compared to the mouse [74] has made the rat an optimal animal model for the investigation of human diseases, such as infectious and autoimmunity diseases, or for toxicology and drug development. Moreover, the rat has other advantages compared to the mouse like for instance the bigger size or the higher learning capacity that make it a convenient research animal model. Nevertheless, the impossibility for many years to generate authentic rat ESCs has given the mouse a clear advantage over the rat as a model for biomedical research.

**Figure 10:**
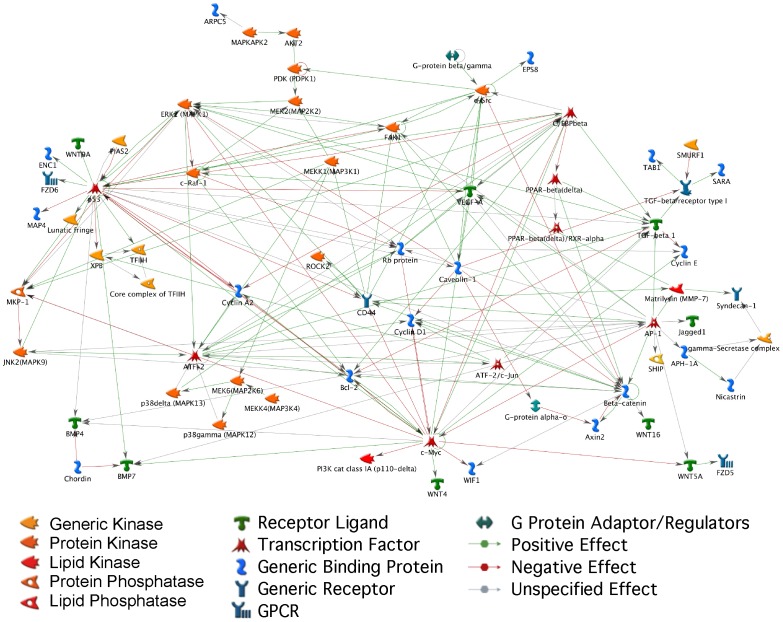
Network of selected differentially expressed genes between mouse and rat in the comparison blastocyst versus morula. Network of selected genes that show different expression patterns in mouse and rat in the comparison blastocyst versus morula. The genes come from the pathway and family gene lists (Table S3 and S4), the graph has been created with GeneGo network editor tool.

With this study we aimed at the identification of differences at the transcriptional level between the mouse and the rat during the embryo development in which the ICM cells are formed, since they represent the source of ESCs derivation. The differential regulation of critical genes could represent the starting point for analyzing their function *in vitro* in mouse and rat ESCs. Furthermore, this knowledge could be critical for the improvement of the derivation and maintenance of rat ESCs. Although recently rat ESCs have been generated [3], [4] there are still many questions open. A broader knowledge on the molecular mechanisms that occur in rat ESCs would improve the efficiency of establishing stable authentic pluripotent rat ESCs and therefore it would facilitate the generation via gene targeting of transgenic rat models, which are indispensable for the biomedical research. This is the first study that investigated the gene expression changes during the transition from morula to blastocyst in the rat preimplantation development. Moreover, our study represents a new example of statistical approach for cross species analysis that could be applicable also for other species. The so-obtained data allows highlighting the species-specific behavior of genes within important pathways and families through the creation of own gene networks. An example of network of genes that behave in a different way in mouse and rat is presented in Figure 10.

We found in our study a number of differentially expressed genes which could be relevant for improving the derivation and maintenance of pluripotent ESCs from the rat. Even though most of the biological processes active during the switch between morula to blastocyst in both species were very similar (Figure S1), a number of differences could be observed in the regulation of specific pathways. Of particular interest is the differential regulation of the Wnt-, Notch- and LIF/Stat3-pathway components. An interest observation for the mouse is that the control of these pathways occurs through the activation of inhibitory components. An example is the high expression of inhibitory components like Maml1 and Tle2/4 to counteract the high Notch1 expression (Figure 3). In the rat this pathway is rather regulated at transcriptional level by directly reducing the expression of Notch1. Also other important genes like β-catenin, *Bmp4*, or *Smad4* show important expression differences. Some of the genes were previously identified to be important factors in the maintenance of pluripotency in ESCs, like for example *Sox2* or *Stat3*, or play a role in reprogramming somatic cells to pluripotency like *c-Myc*, *Klf4* and *p53*.

In summary, this study identified within important signaling pathways interesting candidates differentially expressed in the mouse and in the rat preimplantation embryos. We believe that these differences observed *in vivo* might reflect also the differences observed between the mouse and the rat ESCs, for instance in their derivation efficiency and maintenance. Further analyses are needed in order to clarify which roles do they have in ESCs, and furthermore if they might represent new pluripotency factors. We believe that this study represents a good starting point for further analysis aimed at the specific identification of new factors related to pluripotency in both the species.

## Materials and Methods

### Mouse and Rat Strains

Mouse: Hybrid B6D2F1 mice (female C57BL/6×male DBA/2). Rat: Outbreed Wistar Han rats. Animals were housed under controlled lighting (lights on at 0600–1800 h), temperature (23±2°C) and humidity (50±5%), with free access to food and water. The Veterinary Office of the Canton of Zurich, Switzerland approved all animal experiments. Housing and experimental procedures were in accordance with the Swiss animal protection law and conformed to the European Convention for the protection of vertebrate animals used for experimental and other scientific purposes (Council of Europe no. 123, Strasbourg 1985).

### Collection of Mouse Embryos

3 to 5 weeks old B6D2F1 females were superovulated with 5IU pregnant mare’s serum gonadotropin (PMSG) (Folligon® Intervet) and 48 h later, with 5IU human chronicle gonadotropin (hCG) (Chorulon® Intervet). Superovulated females were mated with B6D2F1 males for 24 h and then housed separately to the males. Upon isolation morula and blastocyst stage embryos were either stored at −80°C for further RNA extraction or immunosurgery was performed for the isolation of the inner cell masses (ICMs).

### Isolation of ICMs by Immunosurgery with Mouse Blastocyst Stage Embryos

Zona pellucida was removed by shortly incubating the blastocysts with Tyrode’s solution (Sigma). Consequently zona-free blastocysts were washed several times with M2 medium (Sigma) and immunosurgery was performed, by incubating the embryos 30 min with rabbit anti-mouse serum (Sigma). Afterwards, embryos were washed several times with DMEM medium (GIBCO) supplemented with 10% of fetal bovine serum. The embryos were then incubated (37C°, 5% CO2) with guinea pig complement serum (Sigma) for another 30 min. Rabbit anti-mouse serum was used 1∶30 diluted in DMEM medium; guinea pig complement serum was diluted 1∶3 in DMEM. The isolated ICMs were stored at −80°C till total RNA was extracted.

### Collection of Rat Embryos

6 to 12 weeks old Wistar females were check for estrus by using the Estrus Cycle Monitor EC40 (Fine Science Tools, Foster City, CA) as previously described [75]. All the positive females were breaded with Wistar males for 24 h and then housed separately to the males. At day E4 of pregnancy Wistar females were sacrificed and morula embryos were isolated and stored for later RNA extraction. Blastocyst embryos were isolated at E4.5 and either collected for further RNA extraction or processed for immunosurgery.

### Immunosurgery with Rat Blastocyst Stage Embryos

After removal of the zona pellucida, blastocysts were incubated for 3 hours (37C°, 5% CO2) in rabbit anti-rat serum (Sigma). Afterwards, embryos were washed several times with DMEM medium (GIBCO) supplemented with 10% of fetal bovine serum and incubated with rat serum (made in-house) for 20 min (37C°, 5% CO2). Rabbit anti-rat serum was used 1∶5 diluted in DMEM medium; rat serum was diluted 1∶5 in DMEM. The isolated ICMs were stored at −80°C till total RNA was extracted.

### Total RNA Extraction From Mouse and Rat Embryos

Till the desired amount of embryos was reached, embryos were stored in RLT buffer supplemented with 1% of β-mercaptoethanol (first lyses buffer of the RNeasy Micro Kit (QIAGEN) protocol) at −80°C. Two pools of embryos for each developmental stage where formed (Figure 1C). Total RNA was extracted by using RNeasy Micro Kit (QIAGEN) according to the manufactures’ recommended protocols. All RNA samples were kept at −80°C till they were processed for microarray hybridization.

### Mouse and Rat Microarray Experiment Description

#### cRNA preparation

The quality of the isolated RNA was determined with a NanoDrop ND 1000 (NanoDrop Technologies, Delaware, USA) and a Bioanalyzer 2100 (Agilent, Waldbronn, Germany). The cDNA was prepared from total RNA using a primer mix and reverse transcriptase (RT) (WTOvation Pico System, NuGEN, 3300-12). The primers have a DNA portion that hybridizes either to the 5′ portion of the poly (A) sequence or randomly across the transcript. SPIA amplification, a linear isothermal DNA amplification process, was used to prepare single-stranded cDNA in the antisense direction of the mRNA starting material. Single-stranded cDNA quality and quantity was determined using NanoDrop ND 1000 and Bioanalyzer 2100. Fragmented and biotin-labeled single-stranded cDNA targets were generated with the FL-Ovation cDNA Biotin Module V2 (NuGEN, 4200-12).

#### Array hybridization

Biotin-labeled single-stranded cDNA targets (5 µg) were mixed in 220 µl of Hybridization Mix (Affymetrix Inc., P/N 900720) containing a Hybridization Controls and Control Oligonucleotide B2 (Affymetrix Inc., P/N 900454). The six mouse samples were hybridized to GeneChip® Mouse Genome 430 2.0 arrays for 18 h at 45°C the same procedure was used for the six rat samples that were hybridized to GeneChip® Rat Genome 230 2.0 arrays. Arrays were then washed using an Affymetrix Fluidics Station 450 FS450 0004 protocol. An Affymetrix GeneChip Scanner 3000 (Affymetrix Inc.) was used to measure the fluorescent intensity emitted by the labeled target.

### Processing of Microarray Data

Raw data processing was performed using the Affymetrix AGCC software. After hybridization and scanning, probe cell intensities were calculated and saved in appropriate CEL files. The CEL files have been processed by scripts in R, prepared with Bioconductor [76] libraries: affy, simpleaffy, limma, gplots and biomaRt. Mouse CEL files have been grouped for summarization with Brainarray [77] custom CDF file Mouse4302_Mm_ENSG (16860 gene product summaries), while rat CEL files had the probes grouped by Rat2302_Rn_ENSG CDF file (11462 gene product summaries). Summarization was performed with standard RMA algorithm [78].

### Mapping of Genes between the Species

The table of mouse-rat orthologs, labeled with Ensembl gene identifiers, has been prepared with biomart interface (http://biomart.org) to Ensembl database [79] (Ensembl genes v60, mouse genome NCBIM 37, rat genome RGSC 3.4). All the mouse genes present in the Brainarray summary file have been translated to rat orthologs, resulting in the translation table that includes 13′139 mouse genes (ca 77.9% of those in the array summary) and 9′083 rat genes (79.2% of those in the array summary). The table has been used to map mouse genes to rat orthologs and *vice versa*.

### Selection of Gene Sets from Pathways and Families

The pathways and the genes included in them have been extracted from Metacore database via GeneGo tool (Thomson Reuters, http://portal.genego.com, [80]). For all the pathways, a list of genes with Ensembl identifiers has been extracted and the genes that have mouse-rat ortholog pairs in the mapping table have been selected. The pathways (groups of genes belonging to the same pathway map in GeneGo Metacore database) and gene families have been selected on the basis of the relevance to various developmental processes. All the analyses have been repeated for the groups of genes belonging to the selected pathways and families. For the graphical overview of the sample processing and data analysis see Figure 1D.

### Discovering Patterns of Similarities and Differences within Groups

For all the pairs of tissues (blastocyst versus morula, ICM versus blastocyst, and ICM versus morula), the fold changes have been calculated for both: mouse and rat separately. To find the genes that have expression characteristics similar or different in each of the pairs of samples the heuristics have been defined. All the fold changes described in the paper are the values after log2 (sometimes called also log2 ratio in the literature) for the sake of symmetry of the fold change distribution and easier tracing the direction of expression changes. The log2 values are also a typical outcome of the algorithms summarizing Affymetrix microarray measurements (eg. RMA). As similar are regarded genes with the difference of fold change smaller than a specific threshold (0.2 on log2 scale). On the scatterplots these genes are marked with red. As different are marked those that have the absolute difference of fold changes bigger than a threshold of 0.4, but also having both mouse and rat fold changes absolute value bigger than a threshold of 0.2 - to choose the genes that are not on the fold change scatterplot diagonal, but excluding the genes not differentially expressed in one of the specie. Genes that satisfy the “differential inter-species fold change conditions” are marked green. The rest of genes that do not fulfill “different” or “similar” condition, are marked plain black on the scatterplot.

“similar” pattern genes (red):



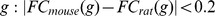



“different” pattern genes (green):



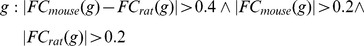



## Supporting Information

Figure S1 **Most significantly enriched Gene Ontology Biological Process terms according to GeneGo.** The lists of genes counted for the enrichment are those that have been used for the Venn diagram with 1.5 fold change differences.
(TIF)

Figure S2 **Cross species analysis of the genes in the p53 pathways. A.** Fold change scatterplots. Cross species comparison of the fold changes of the genes of the pathway “Transcription, p53 signaling pathway” from GeneGo (see also Table S3). The data were analyzed as described in Figure 3A. **B.** Expression signal profile plots. Expression pattern analysis of 4 genes from the p53 pathway. Mouse: blue; Rat: red; MO: Morula; ICM: Inner cell mass; BL: Blastocyst.
(TIF)

Figure S3 **Cross species analysis of PI3K/AKT and MAPK cascades. A.** Fold change scatterplots. Cross species comparison of the fold changes of the genes in the pathway “Development, growth hormone signaling via PI3K/AKT and MAPK cascades” from GeneGo (see also Table S3). The data were analyzed as described in Figure 3A. **B.** Expression signal profile plots. Expression pattern of 6 selected genes. Mouse: blue; Rat: red; MO: Morula; ICM: Inner cell mass; BL: Blastocyst.
(TIF)

Figure S4 **Cross species analysis of factors involved in the regulation of cell proliferation. A.** Fold change scatterplots. Cross species comparison of the fold changes of the genes in the pathway “Development, SSTR2 in regulation of cell proliferation” from GeneGo (see also Table S3). The data were analyzed as described in Figure 3A. **B.** Expression signal profile plots. Expression pattern of 3 selected genes. Mouse: blue; Rat: red; MO: Morula; ICM: Inner cell mass; BL: Blastocyst.
(TIF)

Figure S5 **Cross species analysis of the Wnt pathway. A.** Fold change scatterplots. Cross species comparison of the fold changes of the genes in the pathway “Development, WNT signaling pathway. Part 2” from GeneGo (see also Table S3). The data were analyzed as described in Figure 3A. **B.** Expression signal profile plots. Expression pattern of 6 selected genes. Mouse: blue; Rat: red; MO: Morula; ICM: Inner cell mass; BL: Blastocyst.
(TIF)

Figure S6 **Cross species analysis of apoptotic processes. A.** Fold change scatterplots. Cross species comparison of the fold changes of the genes in the pathway “Apoptosis and survival, NGF signaling pathway” from GeneGo (see also Table S3). The data were analyzed as described in Figure 3A. **B.** Expression signal profile plots. Expression pattern of 4 selected genes. Mouse: blue; Rat: red; MO: Morula; ICM: Inner cell mass; BL: Blastocyst.
(TIF)

Figure S7 **Cross species analysis of apoptotic processes.** **A.** Fold change scatterplots. Cross species comparison of the fold changes of the genes in the pathway “Apoptosis and survival, Apoptotic Activin A signaling” from GeneGo (see also Table S3). The data were analyzed as described in Figure 3A. **B.** Expression signal profile plots. Expression pattern of 5 selected genes. Mouse: blue; Rat: red; MO: Morula; ICM: Inner cell mass; BL: Blastocyst.
(TIF)

Figure S8 **Cross species analysis of MAPK family.** Fold change scatterplots. Scatterplots of the fold changes measured in the three comparisons for 12 members of the MAPK family in the mouse and in the rat. The complete list of all the genes analyzed as well as their fold changes are reported in Table S4.
(TIF)

Table S1 **Mouse Venn diagram 1.5 fold change gene list. A.** ICM versus Blastocyst, **B.** Blastocyst versus Morula, **C**. ICM versus Morula.
(XLS)

Table S2 **Rat Venn diagram 1.5 fold change gene list. A.** ICM versus Blastocyst, **B.** Blastocyst versus Morula, **C**. ICM versus Morula.
(XLS)

Table S3 **Pathway analysis.** List of genes and fold changes in the mouse and in the rat for the three comparisons (blastocyst versus morula, ICM versus blastocyst, ICM versus morula).
(XLS)

Table S4 **Gene family analysis.** List of genes and fold changes in the mouse and in the rat for the three comparisons (blastocyst versus morula, ICM versus blastocyst, ICM versus morula).
(XLS)

Table S5 **Summary of differentially regulated genes between mouse and rat in the pathways analyzed.**
(XLS)

## References

[1] Evans MJ, Kaufman MH (1981) Establishment in culture of pluripotential cells from mouse embryos. Nature 292: 154–156. 7242681 10.1038/292154a0

[2] Martin GR (1981) Isolation of a pluripotent cell line from early mouse embryos cultured in medium conditioned by teratocarcinoma stem cells. Proc Natl Acad Sci U S A 78: 7634–7638. 6950406 10.1073/pnas.78.12.7634 PMC349323

[3] Buehr M, Meek S, Blair K, Yang J, Ure J, et al (2008) Capture of authentic embryonic stem cells from rat blastocysts. Cell 135: 1287–1298. 19109897 10.1016/j.cell.2008.12.007

[4] Li P, Tong C, Mehrian-Shai R, Jia L, Wu N, et al (2008) Germline competent embryonic stem cells derived from rat blastocysts. Cell 135: 1299–1310. 19109898 10.1016/j.cell.2008.12.006 PMC2735113

[5] Ying Q-L, Wray J, Nichols J, Batlle-Morera L, Doble B, et al (2008) The ground state of embryonic stem cell self-renewal. Nature 453: 519–523. 18497825 10.1038/nature06968 PMC5328678

[6] Nichols J, Silva J, Roode M, Smith A (2009) Suppression of Erk signalling promotes ground state pluripotency in the mouse embryo. Development 136: 3215–3222. 19710168 10.1242/dev.038893 PMC2739140

[7] Stewart CL, Kaspar P, Brunet LJ, Bhatt H, Gadi I, et al (1992) Blastocyst implantation depends on maternal expression of leukaemia inhibitory factor. Nature 359: 76–79. 1522892 10.1038/359076a0

[8] Niwa H, Burdon T, Chambers I, Smith A (1998) Self-renewal of pluripotent embryonic stem cells is mediated via activation of STAT3. Genes Dev 12: 2048–2060. 9649508 10.1101/gad.12.13.2048 PMC316954

[9] Matsuda T, Nakamura T, Nakao K, Arai T, Katsuki M, et al (1999) STAT3 activation is sufficient to maintain an undifferentiated state of mouse embryonic stem cells. Embo J 18: 4261–4269. 10428964 10.1093/emboj/18.15.4261 PMC1171502

[10] Cinelli P, Casanova EA, Uhlig S, Lochmatter P, Matsuda T, et al (2008) Expression profiling in transgenic FVB/N embryonic stem cells overexpressing STAT3. BMC Dev Biol 8: 57. 18500982 10.1186/1471-213X-8-57 PMC2409313

[11] Ladd AN, Charlet N, Cooper TA (2001) The CELF family of RNA binding proteins is implicated in cell-specific and developmentally regulated alternative splicing. Mol Cell Biol 21: 1285–1296. 11158314 10.1128/MCB.21.4.1285-1296.2001 PMC99581

[12] Yang DH, Smith ER, Cai KQ, Xu XX (2009) C-Fos elimination compensates for disabled-2 requirement in mouse extraembryonic endoderm development. Dev Dyn 238: 514–523. 19191218 10.1002/dvdy.21856 PMC2743073

[13] Asher G, Lotem J, Cohen B, Sachs L, Shaul Y (2001) Regulation of p53 stability and p53-dependent apoptosis by NADH quinone oxidoreductase 1. Proc Natl Acad Sci U S A 98: 1188–1193. 11158615 10.1073/pnas.021558898 PMC14730

[14] Levine AJ (1997) p53, the cellular gatekeeper for growth and division. Cell 88: 323–331. 9039259 10.1016/s0092-8674(00)81871-1

[15] Vogelstein B, Lane D, Levine AJ (2000) Surfing the p53 network. Nature 408: 307–310. 11099028 10.1038/35042675

[16] Lowe SW, Schmitt EM, Smith SW, Osborne BA, Jacks T (1993) p53 is required for radiation-induced apoptosis in mouse thymocytes. Nature 362: 847–849. 8479522 10.1038/362847a0

[17] Artavanis-Tsakonas S, Rand MD, Lake RJ (1999) Notch signaling: cell fate control and signal integration in development. Science 284: 770–776. 10221902 10.1126/science.284.5415.770

[18] Kadesch T (2004) Notch signaling: the demise of elegant simplicity. Curr Opin Genet Dev 14: 506–512. 15380241 10.1016/j.gde.2004.07.007

[19] Ma G, Li T, Price DL, Wong PC (2005) APH-1a is the principal mammalian APH-1 isoform present in gamma-secretase complexes during embryonic development. J Neurosci 25: 192–198. 15634781 10.1523/JNEUROSCI.3814-04.2005 PMC6725209

[20] Giagtzoglou N, Alifragis P, Koumbanakis KA, Delidakis C (2003) Two modes of recruitment of E(spl) repressors onto target genes. Development (Cambridge, England) 130: 259–270. 10.1242/dev.00206 12466194

[21] Lowell S, Benchoua A, Heavey B, Smith AG (2006) Notch promotes neural lineage entry by pluripotent embryonic stem cells. PLoS Biol 4: e121. 16594731 10.1371/journal.pbio.0040121 PMC1431581

[22] Nagy A, Gertsenstein M, Vintersten K, Behringer R (2003) Manipulating the mouse embryo: A laboratory Manual. Cold Spring Harbor, New York: Cold Spring Harbor Laboratory Press.

[23] Ciemerych MA, Sicinski P (2005) Cell cycle in mouse development. Oncogene 24: 2877–2898. 15838522 10.1038/sj.onc.1208608

[24] Savatier P, Huang S, Szekely L, Wiman KG, Samarut J (1994) Contrasting patterns of retinoblastoma protein expression in mouse embryonic stem cells and embryonic fibroblasts. Oncogene 9: 809–818. 8108123

[25] Harbour JW, Dean DC (2000) The Rb/E2F pathway: expanding roles and emerging paradigms. Genes Dev 14: 2393–2409. 11018009 10.1101/gad.813200

[26] Carrano AC, Eytan E, Hershko A, Pagano M (1999) SKP2 is required for ubiquitin-mediated degradation of the CDK inhibitor p27. Nat Cell Biol 1: 193–199. 10559916 10.1038/12013

[27] Somlyo AP, Somlyo AV (1994) Signal transduction and regulation in smooth muscle. Nature 372: 231–236. 7969467 10.1038/372231a0

[28] Moussavi RS, Kelley CA, Adelstein RS (1993) Phosphorylation of vertebrate nonmuscle and smooth muscle myosin heavy chains and light chains. Mol Cell Biochem 127–128: 219–227. 10.1007/BF01076773 7935353

[29] Kimura K, Ito M, Amano M, Chihara K, Fukata Y, et al (1996) Regulation of myosin phosphatase by Rho and Rho-associated kinase (Rho-kinase). Science 273: 245–248. 8662509 10.1126/science.273.5272.245

[30] Amano M, Ito M, Kimura K, Fukata Y, Chihara K, et al (1996) Phosphorylation and activation of myosin by Rho-associated kinase (Rho-kinase). J Biol Chem 271: 20246–20249. 8702756 10.1074/jbc.271.34.20246

[31] Yamakita Y, Yamashiro S, Matsumura F (1994) In vivo phosphorylation of regulatory light chain of myosin II during mitosis of cultured cells. J Cell Biol 124: 129–137. 8294496 10.1083/jcb.124.1.129 PMC2119899

[32] Totsukawa G, Yamakita Y, Yamashiro S, Hosoya H, Hartshorne DJ, et al (1999) Activation of myosin phosphatase targeting subunit by mitosis-specific phosphorylation. J Cell Biol 144: 735–744. 10037794 10.1083/jcb.144.4.735 PMC2132942

[33] Lemaitre JM, Buckle RS, Mechali M (1996) c-Myc in the control of cell proliferation and embryonic development. Adv Cancer Res 70: 95–144. 8902055 10.1016/s0065-230x(08)60873-8

[34] Takahashi K, Yamanaka S (2006) Induction of pluripotent stem cells from mouse embryonic and adult fibroblast cultures by defined factors. Cell 126: 663–676. 16904174 10.1016/j.cell.2006.07.024

[35] Smith AG, Heath JK, Donaldson DD, Wong GG, Moreau J, et al (1988) Inhibition of pluripotential embryonic stem cell differentiation by purified polypeptides. Nature 336: 688–690. 3143917 10.1038/336688a0

[36] Ying QL, Nichols J, Chambers I, Smith A (2003) BMP induction of Id proteins suppresses differentiation and sustains embryonic stem cell self-renewal in collaboration with STAT3. Cell 115: 281–292. 14636556 10.1016/s0092-8674(03)00847-x

[37] Li W, Wei W, Zhu S, Zhu J, Shi Y, et al (2009) Generation of rat and human induced pluripotent stem cells by combining genetic reprogramming and chemical inhibitors. Cell stem cell 4: 16–19. 19097958 10.1016/j.stem.2008.11.014

[38] Hamanaka S, Yamaguchi T, Kobayashi T, Kato-Itoh M, Yamazaki S, et al (2011) Generation of germline-competent rat induced pluripotent stem cells. PLoS ONE 6: e22008. 21789202 10.1371/journal.pone.0022008 PMC3137610

[39] Shen Y, Shi C, Wei W, Yu W, Li W, et al (2011) The heterogeneity and dynamic equilibrium of rat embryonic stem cells. Cell research 21: 1143–1147. 21670743 10.1038/cr.2011.98 PMC3193490

[40] Kitisin K, Saha T, Blake T, Golestaneh N, Deng M, et al (2007) Tgf-Beta signaling in development. Sci STKE 2007: cm1. 17699101 10.1126/stke.3992007cm1

[41] Wu MY, Hill CS (2009) Tgf-beta superfamily signaling in embryonic development and homeostasis. Dev Cell 16: 329–343. 19289080 10.1016/j.devcel.2009.02.012

[42] Yi F, Pereira L, Hoffman JA, Shy BR, Yuen CM, et al.. (2011) Opposing effects of Tcf3 and Tcf1 control Wnt stimulation of embryonic stem cell self-renewal. Nature cell biology. 10.1038/ncb2283 PMC3129424 21685894

[43] Wray J, Kalkan T, Gomez-Lopez S, Eckardt D, Cook A, et al.. (2011) Inhibition of glycogen synthase kinase-3 alleviates Tcf3 repression of the pluripotency network and increases embryonic stem cell resistance to differentiation. Nature cell biology. 10.1038/ncb2267 PMC3160487 21685889

[44] Kelly KF, Ng DY, Jayakumaran G, Wood GA, Koide H, et al (2011) β-Catenin Enhances Oct-4 Activity and Reinforces Pluripotency through a TCF-Independent Mechanism. Cell stem cell 8: 214–227. 21295277 10.1016/j.stem.2010.12.010 PMC3465368

[45] Drab M, Verkade P, Elger M, Kasper M, Lohn M, et al (2001) Loss of caveolae, vascular dysfunction, and pulmonary defects in caveolin-1 gene-disrupted mice. Science 293: 2449–2452. 11498544 10.1126/science.1062688

[46] Nabi IR, Le PU (2003) Caveolae/raft-dependent endocytosis. J Cell Biol 161: 673–677. 12771123 10.1083/jcb.200302028 PMC2199359

[47] Shatz M, Liscovitch M (2008) Caveolin-1: a tumor-promoting role in human cancer. Int J Radiat Biol 84: 177–189. 18300018 10.1080/09553000701745293

[48] Galbiati F, Volonte D, Brown AM, Weinstein DE, Ben-Ze’ev A, et al (2000) Caveolin-1 expression inhibits Wnt/beta-catenin/Lef-1 signaling by recruiting beta-catenin to caveolae membrane domains. J Biol Chem 275: 23368–23377. 10816572 10.1074/jbc.M002020200

[49] Jho E-h, Zhang T, Domon C, Joo C-K, Freund J-N, et al (2002) Wnt/beta-catenin/Tcf signaling induces the transcription of Axin2, a negative regulator of the signaling pathway. Mol Cell Biol 22: 1172–1183. 11809808 10.1128/MCB.22.4.1172-1183.2002 PMC134648

[50] Lustig B, Jerchow B, Sachs M, Weiler S, Pietsch T, et al (2002) Negative feedback loop of Wnt signaling through upregulation of conductin/axin2 in colorectal and liver tumors. Mol Cell Biol 22: 1184–1193. 11809809 10.1128/MCB.22.4.1184-1193.2002 PMC134640

[51] Fujita J, Crane AM, Souza MK, Dejosez M, Kyba M, et al (2008) Caspase activity mediates the differentiation of embryonic stem cells. Cell stem cell 2: 595–601. 18522852 10.1016/j.stem.2008.04.001 PMC2494585

[52] Goldfarb M (1996) Functions of fibroblast growth factors in vertebrate development. Cytokine Growth Factor Rev 7: 311–325. 9023055 10.1016/s1359-6101(96)00039-1

[53] Dailey L, Ambrosetti D, Mansukhani A, Basilico C (2005) Mechanisms underlying differential responses to FGF signaling. Cytokine Growth Factor Rev 16: 233–247. 15863038 10.1016/j.cytogfr.2005.01.007

[54] Mohammadi M, Olsen SK, Ibrahimi OA (2005) Structural basis for fibroblast growth factor receptor activation. Cytokine Growth Factor Rev 16: 107–137. 15863029 10.1016/j.cytogfr.2005.01.008

[55] Asanoma K, Rumi MAK, Kent LN, Chakraborty D, Renaud SJ, et al (2011) FGF4-dependent stem cells derived from rat blastocysts differentiate along the trophoblast lineage. Dev Biol 351: 110–119. 21215265 10.1016/j.ydbio.2010.12.038 PMC3039089

[56] Wang Y, Wang F, Sun T, Trostinskaia A, Wygle D, et al (2004) Entire mitogen activated protein kinase (MAPK) pathway is present in preimplantation mouse embryos. Dev Dyn 231: 72–87. 15305288 10.1002/dvdy.20114

[57] Mikels AJ, Nusse R (2006) Purified Wnt5a protein activates or inhibits beta-catenin-TCF signaling depending on receptor context. PLoS Biol 4: e115. 16602827 10.1371/journal.pbio.0040115 PMC1420652

[58] Hao J, Li T-G, Qi X, Zhao D-F, Zhao G-Q (2006) WNT/beta-catenin pathway up-regulates Stat3 and converges on LIF to prevent differentiation of mouse embryonic stem cells. Dev Biol 290: 81–91. 16330017 10.1016/j.ydbio.2005.11.011

[59] Frank DA (2007) STAT3 as a central mediator of neoplastic cellular transformation. Cancer Lett 251: 199–210. 17129668 10.1016/j.canlet.2006.10.017

[60] Fu XY, Schindler C, Improta T, Aebersold R, Darnell JE (1992) The proteins of ISGF-3, the interferon alpha-induced transcriptional activator, define a gene family involved in signal transduction. Proc Natl Acad Sci USA 89: 7840–7843. 1502204 10.1073/pnas.89.16.7840 PMC49807

[61] Casanova EA, Shakhova O, Patel SS, Asner IN, Pelczar P, et al (2011) Pramel7 mediates LIF/STAT3-dependent self-renewal in embryonic stem cells. Stem cells (Dayton, Ohio) 29: 474–485. 10.1002/stem.588 21425410

[62] Li M, Sendtner M, Smith A (1995) Essential function of LIF receptor in motor neurons. Nature 378: 724–727. 7501019 10.1038/378724a0

[63] Nakashima K, Wiese S, Yanagisawa M, Arakawa H, Kimura N, et al (1999) Developmental requirement of gp130 signaling in neuronal survival and astrocyte differentiation. J Neurosci 19: 5429–5434. 10377352 10.1523/JNEUROSCI.19-13-05429.1999 PMC6782325

[64] Nichols J, Chambers I, Taga T, Smith A (2001) Physiological rationale for responsiveness of mouse embryonic stem cells to gp130 cytokines. Development (Cambridge, England) 128: 2333–2339. 10.1242/dev.128.12.2333 11493552

[65] Graf U, Casanova EA, Cinelli P (2011) The Role of the Leukemia Inhibitory Factor (LIF) – Pathway in Derivation and Maintenance of Murine Pluripotent Stem Cells. Genes 2: 280–297. 24710148 10.3390/genes2010280 PMC3924847

[66] O’Sullivan LA, Liongue C, Lewis RS, Stephenson SE, Ward AC (2007) Cytokine receptor signaling through the Jak-Stat-Socs pathway in disease. Mol Immunol 44: 2497–2506. 17208301 10.1016/j.molimm.2006.11.025

[67] Fukada T, Hibi M, Yamanaka Y, Takahashi-Tezuka M, Fujitani Y, et al (1996) Two signals are necessary for cell proliferation induced by a cytokine receptor gp130: involvement of STAT3 in anti-apoptosis. Immunity 5: 449–460. 8934572 10.1016/s1074-7613(00)80501-4

[68] Kunath T, Saba-El-Leil MK, Almousailleakh M, Wray J, Meloche S, et al (2007) FGF stimulation of the Erk1/2 signalling cascade triggers transition of pluripotent embryonic stem cells from self-renewal to lineage commitment. Development 134: 2895–2902. 17660198 10.1242/dev.02880

[69] Masui S, Nakatake Y, Toyooka Y, Shimosato D, Yagi R, et al (2007) Pluripotency governed by Sox2 via regulation of Oct3/4 expression in mouse embryonic stem cells. Nat Cell Biol 9: 625–635. 17515932 10.1038/ncb1589

[70] Hall J, Guo G, Wray J, Eyres I, Nichols J, et al (2009) Oct4 and LIF/Stat3 Additively Induce Krüppel Factors to Sustain Embryonic Stem Cell Self-Renewal. Cell stem cell 5: 597–609. 19951688 10.1016/j.stem.2009.11.003

[71] Niwa H, Ogawa K, Shimosato D, Adachi K (2009) A parallel circuit of LIF signalling pathways maintains pluripotency of mouse ES cells. Nature 460: 118–122. 19571885 10.1038/nature08113

[72] Chang M-Y, Kim D, Kim C-H, Kang H-C, Yang E, et al (2010) Direct reprogramming of rat neural precursor cells and fibroblasts into pluripotent stem cells. PLoS ONE 5: e9838. 20352099 10.1371/journal.pone.0009838 PMC2844422

[73] Liao J, Cui C, Chen S, Ren J, Chen J, et al (2009) Generation of induced pluripotent stem cell lines from adult rat cells. Cell stem cell 4: 11–15. 19097959 10.1016/j.stem.2008.11.013

[74] Canzian F (1997) Phylogenetics of the laboratory rat Rattus norvegicus. Genome research 7: 262–267. 9074929 10.1101/gr.7.3.262

[75] Ramos SD, Lee JM, Peuler JD (2001) An inexpensive meter to measure differences in electrical resistance in the rat vagina during the ovarian cycle. J Appl Physiol 91: 667–670. 11457779 10.1152/jappl.2001.91.2.667

[76] Gentleman RC, Carey VJ, Bates DM, Bolstad B, Dettling M, et al (2004) Bioconductor: open software development for computational biology and bioinformatics. Genome Biol 5: R80. 15461798 10.1186/gb-2004-5-10-r80 PMC545600

[77] Dai M, Wang P, Boyd AD, Kostov G, Athey B, et al (2005) Evolving gene/transcript definitions significantly alter the interpretation of GeneChip data. Nucleic Acids Res 33: e175. 16284200 10.1093/nar/gni179 PMC1283542

[78] Irizarry RA, Hobbs B, Collin F, Beazer-Barclay YD, Antonellis KJ, et al (2003) Exploration, normalization, and summaries of high density oligonucleotide array probe level data. Biostatistics 4: 249–264. 12925520 10.1093/biostatistics/4.2.249

[79] Flicek P, Amode MR, Barrell D, Beal K, Brent S, et al (2011) Ensembl 2011. Nucleic Acids Res 39: D800–806. 21045057 10.1093/nar/gkq1064 PMC3013672

[80] Nikolsky Y, Kirillov E, Zuev R, Rakhmatulin E, Nikolskaya T (2009) Functional analysis of OMICs data and small molecule compounds in an integrated “knowledge-based” platform. Methods Mol Biol 563: 177–196. 19597786 10.1007/978-1-60761-175-2_10

