# Supplementary figures and images for: Cross-Species Genome Wide Expression Analysis during Pluripotent Cell Determination in Mouse and Rat Preimplantation Embryos
Source: PLoS One. 2012 Oct 15;7(10):e47107. doi: 10.1371/journal.pone.0047107 (PMC3471948; doi:10.1371/journal.pone.0047107)

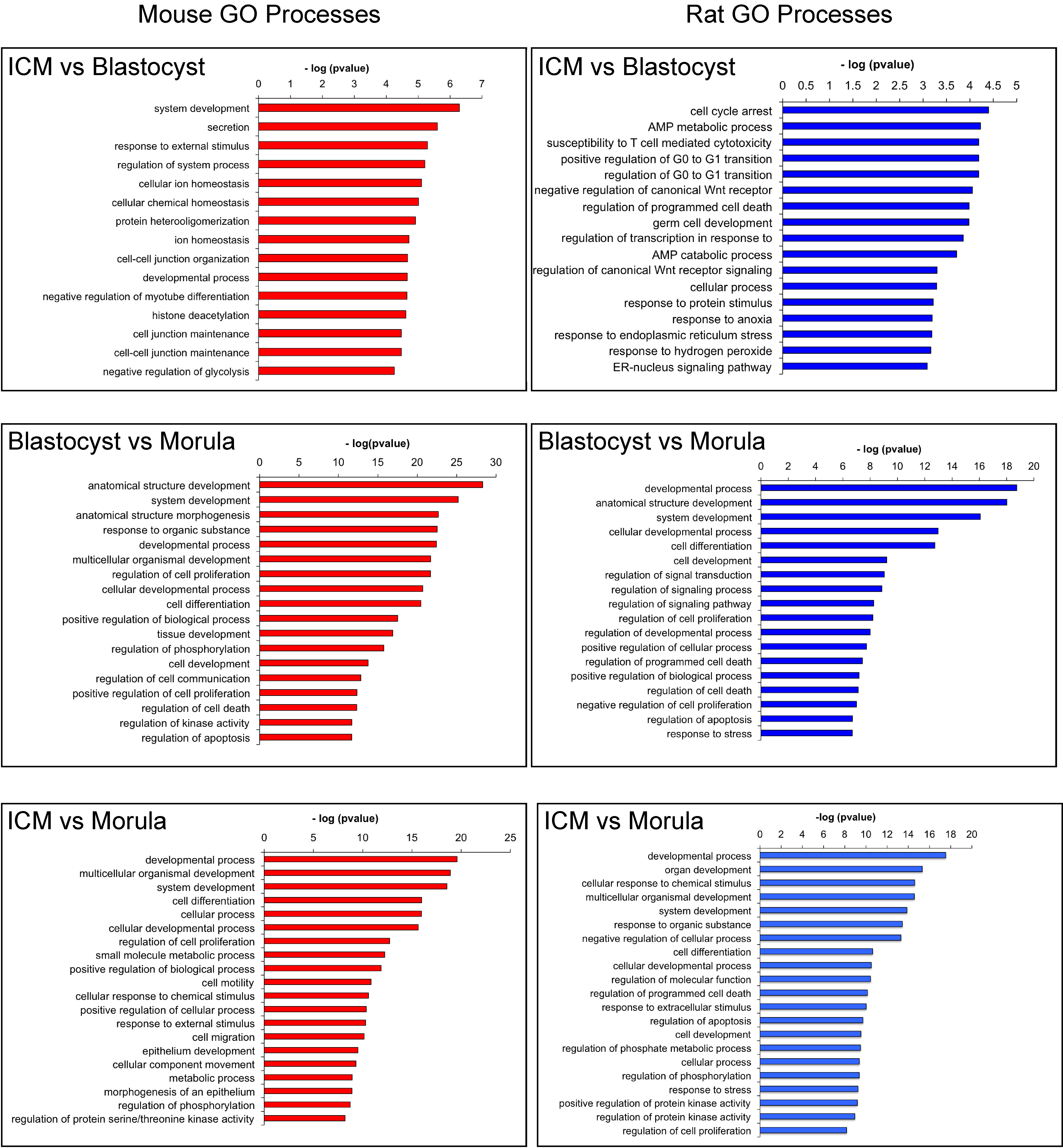

Supplement: Figure S1 — Most significantly enriched Gene Ontology Biological Process terms according to GeneGo. The lists of genes counted for the enrichment are those that have been used for the Venn diagram with 1.5 fold change differences. (TIF) [file pone.0047107.s001.tif]

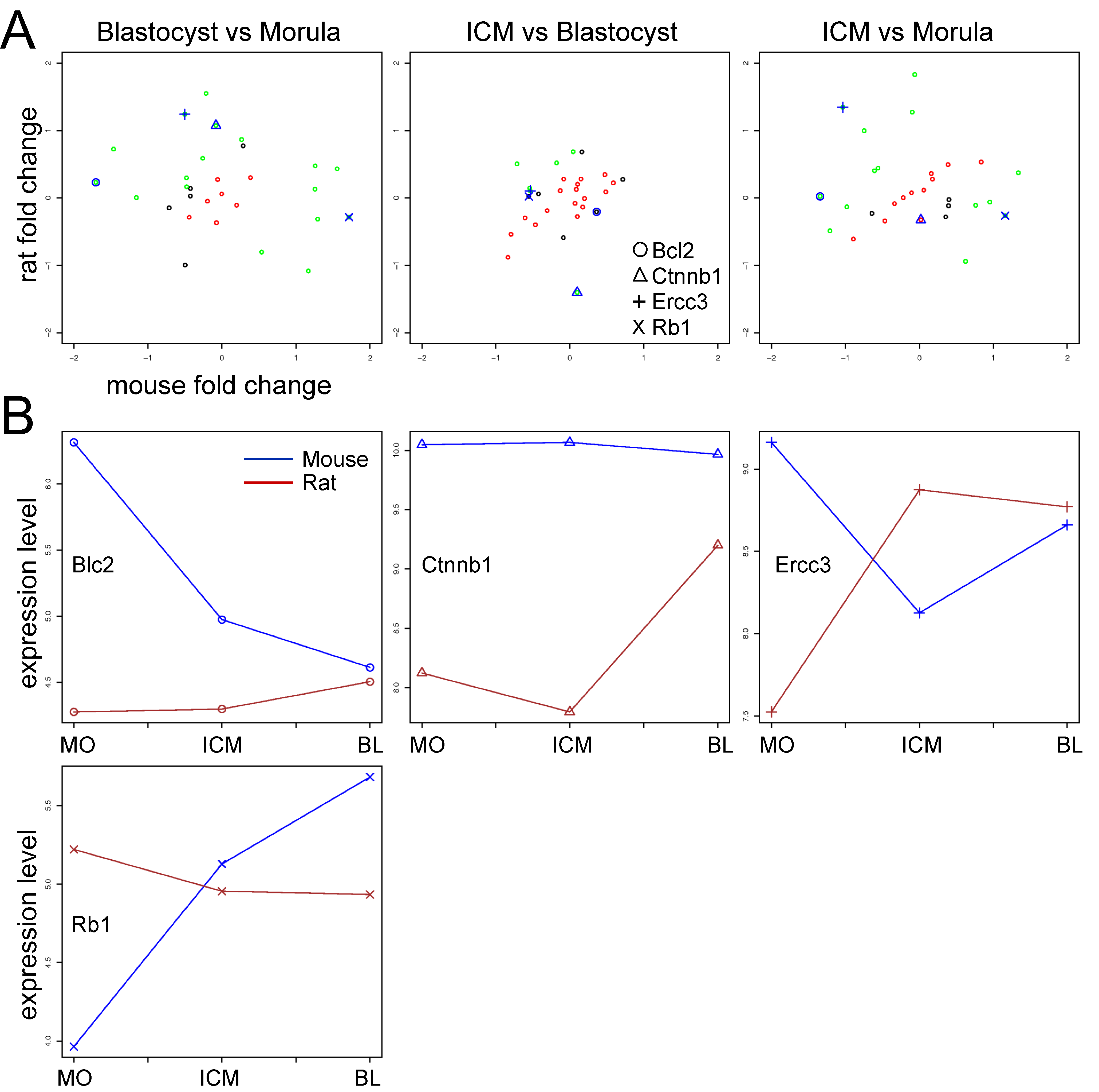

Supplement: Figure S2 — Cross species analysis of the genes in the p53 pathways. A. Fold change scatterplots. Cross species comparison of the fold changes of the genes of the pathway “Transcription, p53 signaling pathway” from GeneGo (see also Table S3). The data were analyzed as described in Figure 3A. B. Expression signal profile plots. Expression pattern analysis of 4 genes from the p53 pathway. Mouse: blue; Rat: red; MO: Morula; ICM: Inner cell mass; BL: Blastocyst. (TIF) [file pone.0047107.s002.tif]

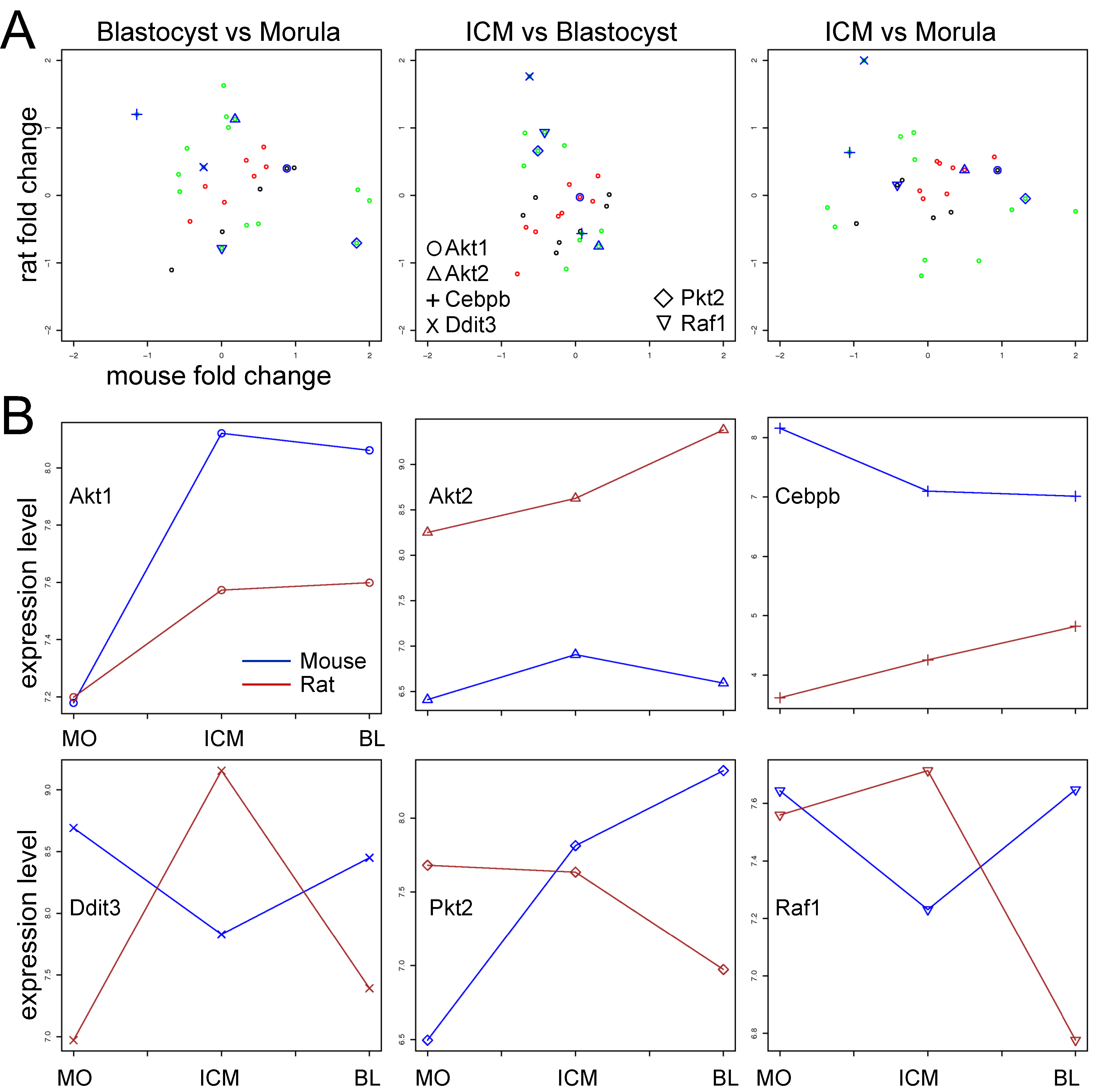

Supplement: Figure S3 — Cross species analysis of PI3K/AKT and MAPK cascades. A. Fold change scatterplots. Cross species comparison of the fold changes of the genes in the pathway “Development, growth hormone signaling via PI3K/AKT and MAPK cascades” from GeneGo (see also Table S3). The data were analyzed as described in Figure 3A. B. Expression signal profile plots. Expression pattern of 6 selected genes. Mouse: blue; Rat: red; MO: Morula; ICM: Inner cell mass; BL: Blastocyst. (TIF) [file pone.0047107.s003.tif]

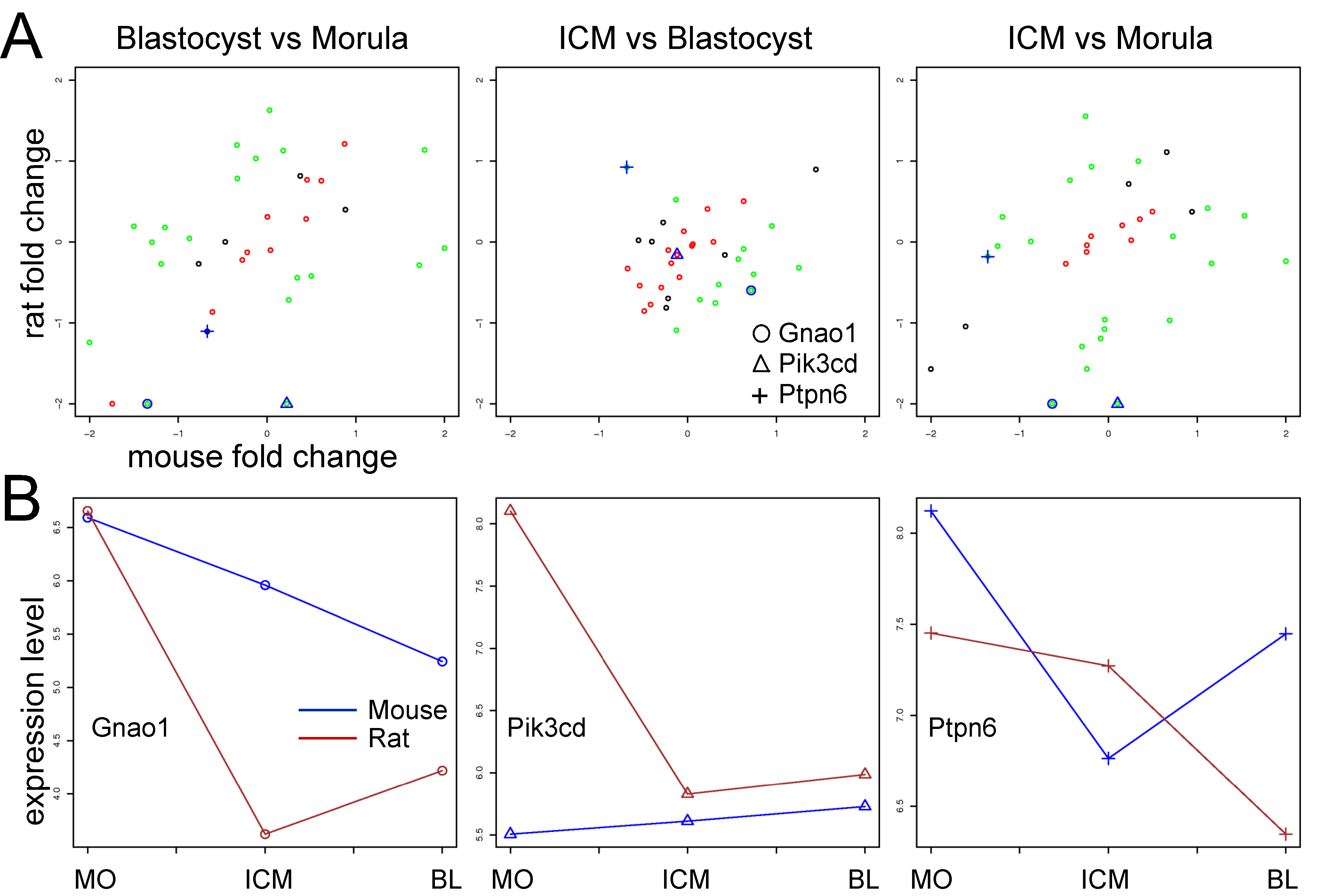

Supplement: Figure S4 — Cross species analysis of factors involved in the regulation of cell proliferation. A. Fold change scatterplots. Cross species comparison of the fold changes of the genes in the pathway “Development, SSTR2 in regulation of cell proliferation” from GeneGo (see also Table S3). The data were analyzed as described in Figure 3A. B. Expression signal profile plots. Expression pattern of 3 selected genes. Mouse: blue; Rat: red; MO: Morula; ICM: Inner cell mass; BL: Blastocyst. (TIF) [file pone.0047107.s004.tif]

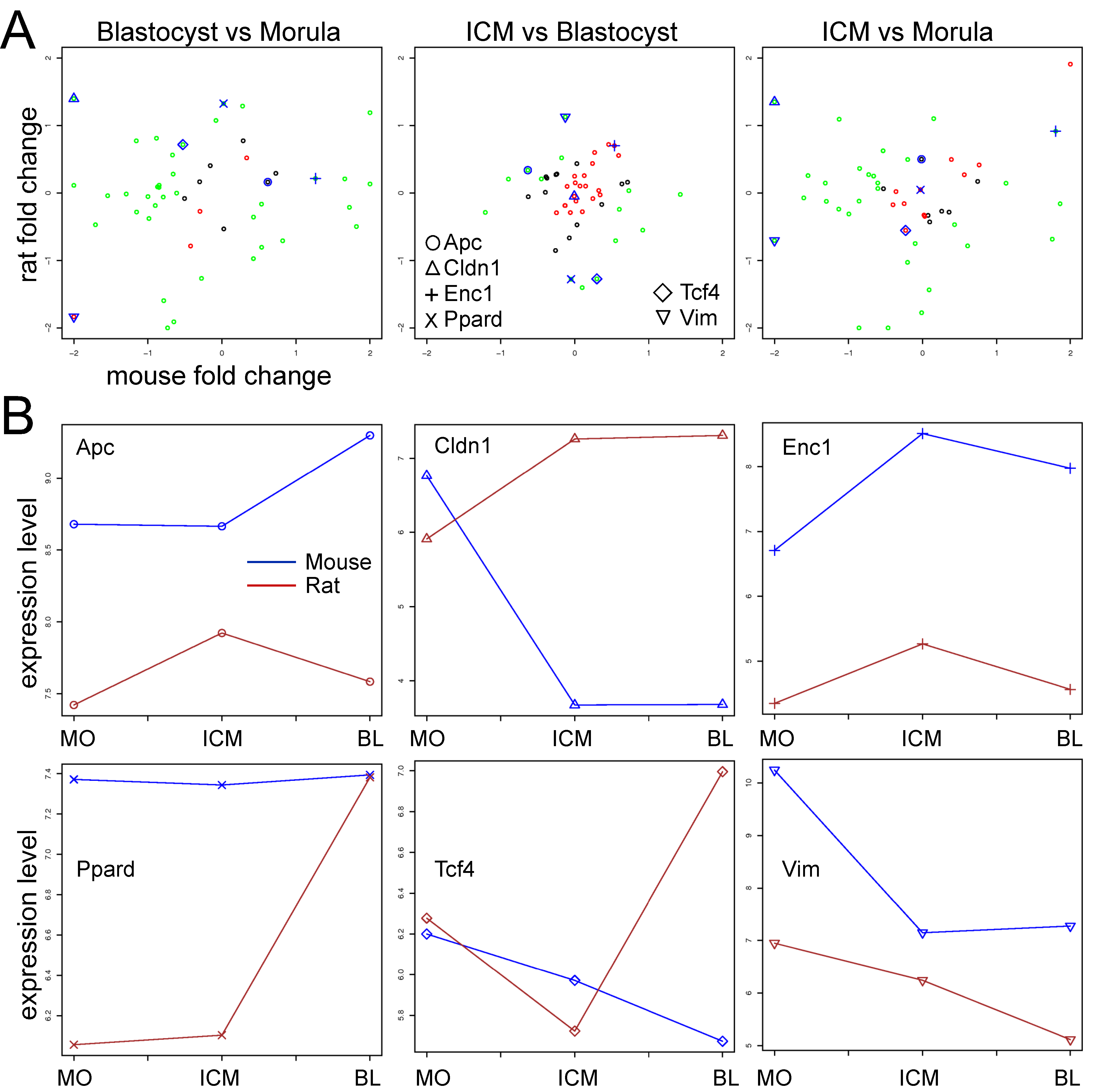

Supplement: Figure S5 — Cross species analysis of the Wnt pathway. A. Fold change scatterplots. Cross species comparison of the fold changes of the genes in the pathway “Development, WNT signaling pathway. Part 2” from GeneGo (see also Table S3). The data were analyzed as described in Figure 3A. B. Expression signal profile plots. Expression pattern of 6 selected genes. Mouse: blue; Rat: red; MO: Morula; ICM: Inner cell mass; BL: Blastocyst. (TIF) [file pone.0047107.s005.tif]

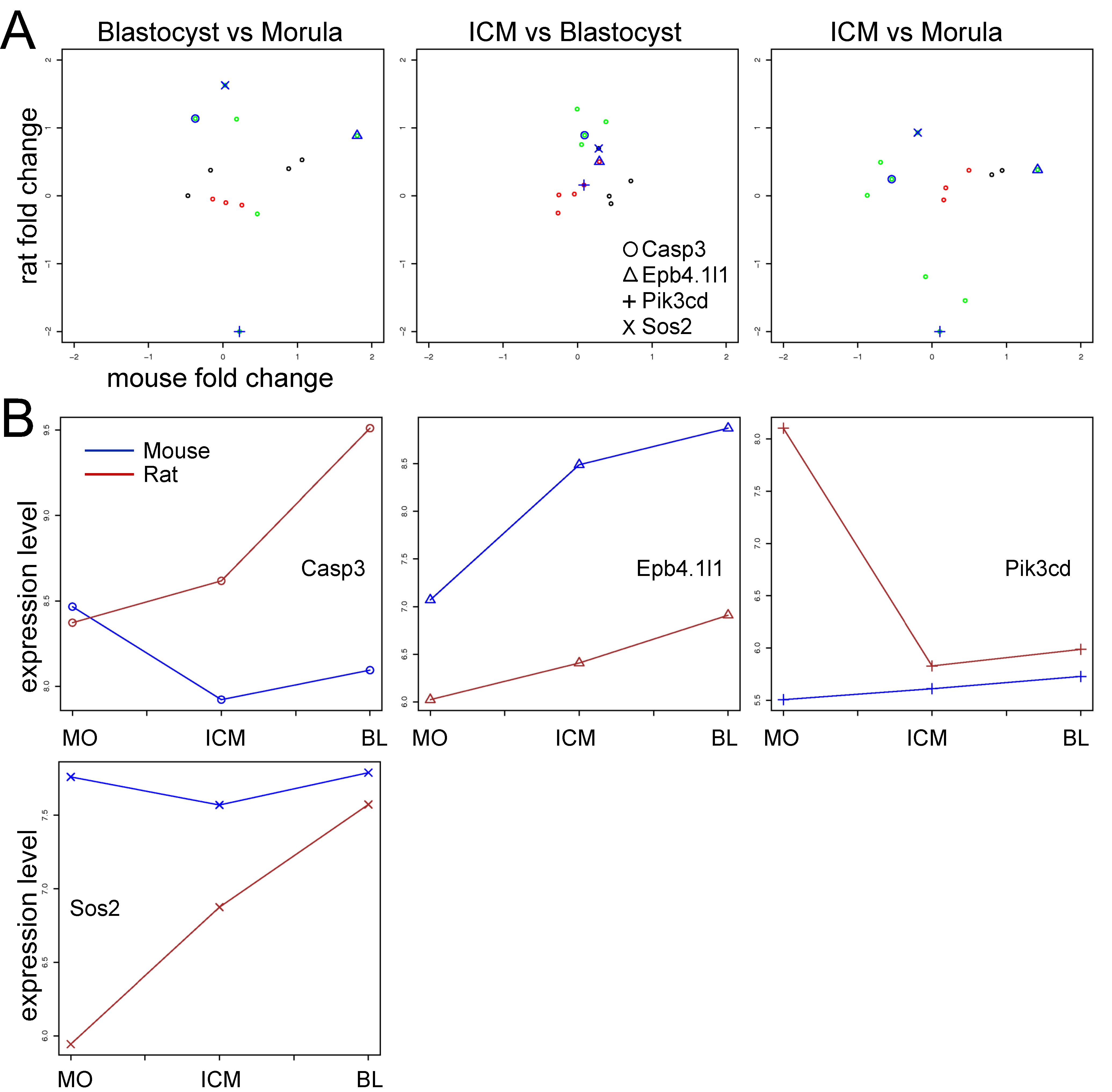

Supplement: Figure S6 — Cross species analysis of apoptotic processes. A. Fold change scatterplots. Cross species comparison of the fold changes of the genes in the pathway “Apoptosis and survival, NGF signaling pathway” from GeneGo (see also Table S3). The data were analyzed as described in Figure 3A. B. Expression signal profile plots. Expression pattern of 4 selected genes. Mouse: blue; Rat: red; MO: Morula; ICM: Inner cell mass; BL: Blastocyst. (TIF) [file pone.0047107.s006.tif]

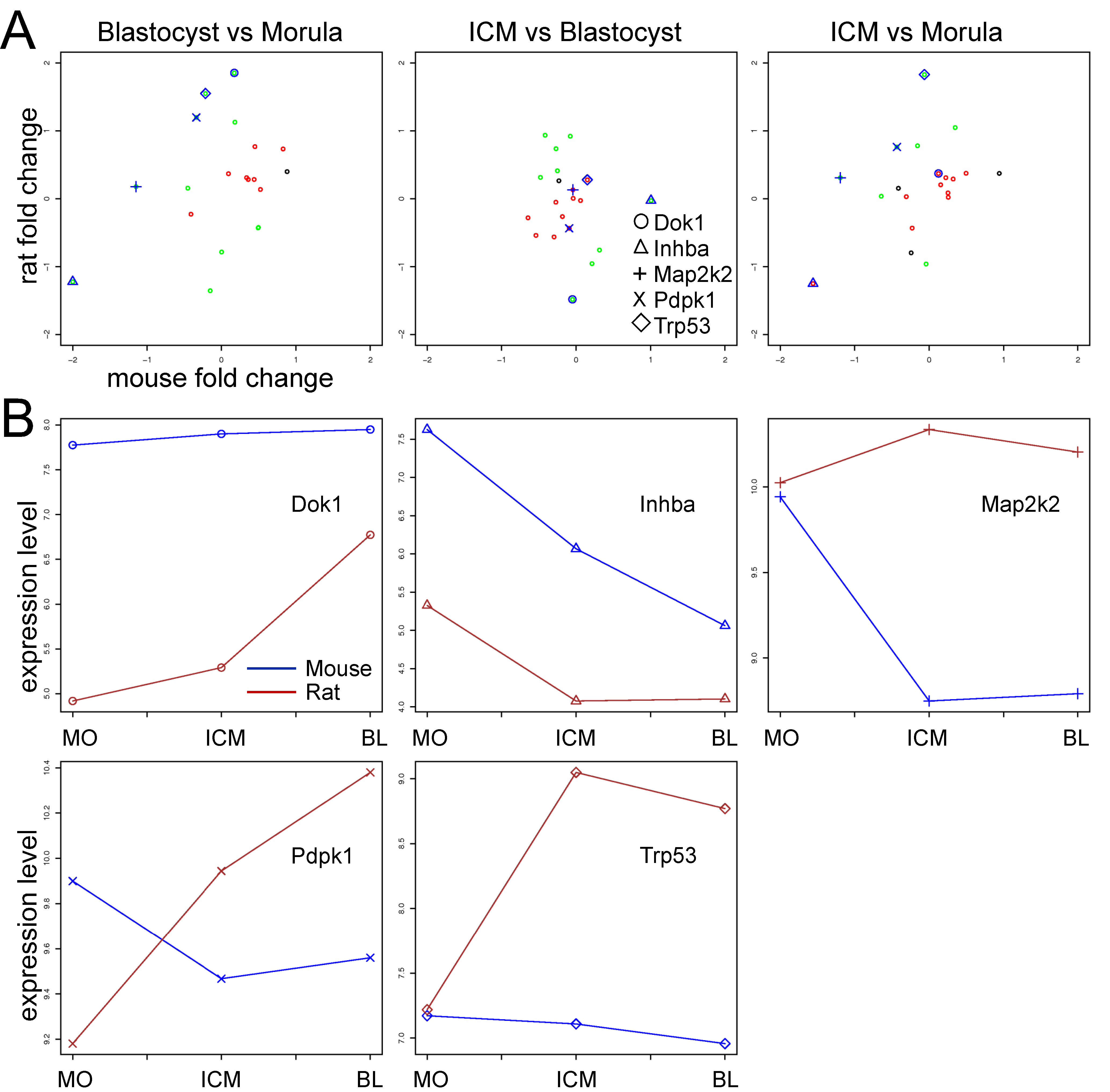

Supplement: Figure S7 — Cross species analysis of apoptotic processes. A. Fold change scatterplots. Cross species comparison of the fold changes of the genes in the pathway “Apoptosis and survival, Apoptotic Activin A signaling” from GeneGo (see also Table S3). The data were analyzed as described in Figure 3A. B. Expression signal profile plots. Expression pattern of 5 selected genes. Mouse: blue; Rat: red; MO: Morula; ICM: Inner cell mass; BL: Blastocyst. (TIF) [file pone.0047107.s007.tif]

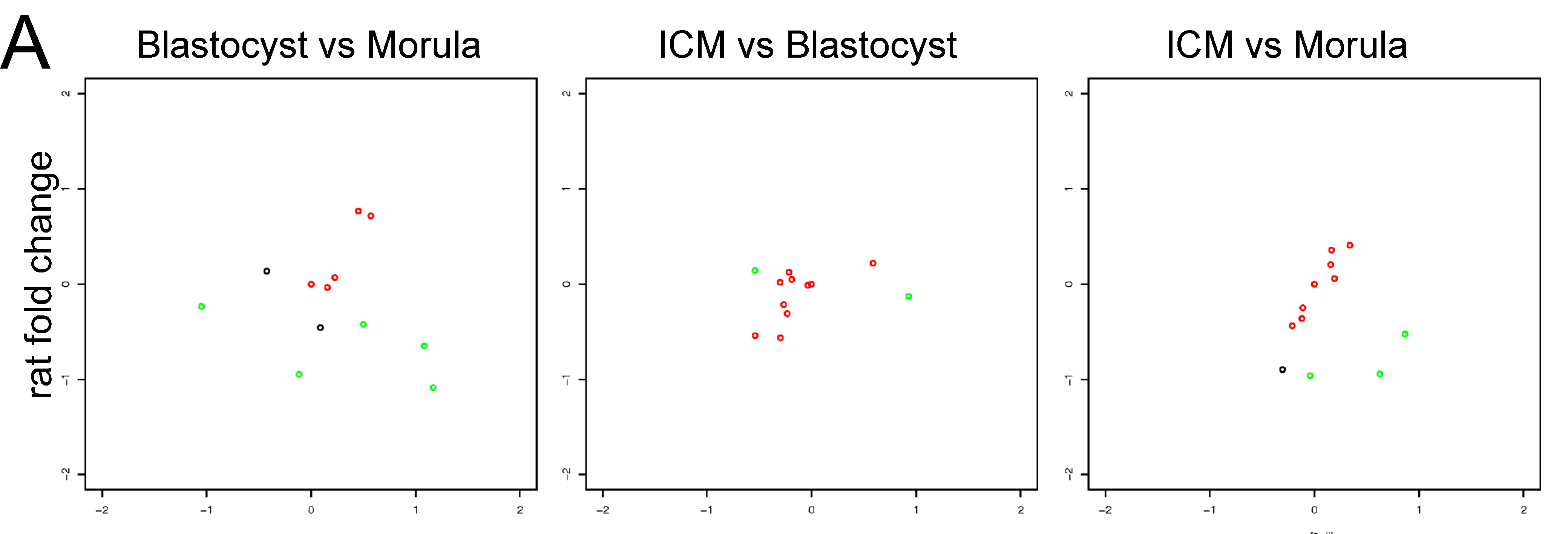

Supplement: Figure S8 — Cross species analysis of MAPK family. Fold change scatterplots. Scatterplots of the fold changes measured in the three comparisons for 12 members of the MAPK family in the mouse and in the rat. The complete list of all the genes analyzed as well as their fold changes are reported in Table S4. (TIF) [file pone.0047107.s008.tif]
